# Type 2 immunity in allergic diseases

**DOI:** 10.1038/s41423-025-01261-2

**Published:** 2025-02-17

**Authors:** Ismail Ogulur, Yasutaka Mitamura, Duygu Yazici, Yagiz Pat, Sena Ardicli, Manru Li, Paolo D’Avino, Carina Beha, Huseyn Babayev, Bingjie Zhao, Can Zeyneloglu, Oliva Giannelli Viscardi, Ozge Ardicli, Ayca Kiykim, Asuncion Garcia-Sanchez, Juan-Felipe Lopez, Li-li Shi, Minglin Yang, Stephan R. Schneider, Stephen Skolnick, Raja Dhir, Urszula Radzikowska, Abhijeet J. Kulkarni, Manal Bel Imam, Willem van de Veen, Milena Sokolowska, Mar Martin-Fontecha, Oscar Palomares, Kari C. Nadeau, Mubeccel Akdis, Cezmi A. Akdis

**Affiliations:** 1 Swiss Institute of Allergy and Asthma Research (SIAF), University of Zurich, Davos, Switzerland; 2 Department of Genetics, Faculty of Veterinary Medicine, Bursa Uludag University, Bursa, Turkey; 3 Division of Food Processing, Milk and Dairy Products Technology Program, Karacabey Vocational School, Bursa Uludag University, Bursa, Turkey; 4 Department of Pediatrics, Division of Pediatric Allergy and Immunology, Cerrahpasa School of Medicine, Istanbul University-Cerrahpasa, Istanbul, Turkey; 5 Department of Biomedical and Diagnostic Science, School of Medicine, University of Salamanca, Salamanca, Spain; 6 Department of Otolaryngology-Head and Neck Surgery, Tongji Hospital, Tongji Medical College, Huazhong University of Science and Technology, Wuhan, P.R. China; 7 Seed Health Inc., Los Angeles, CA USA; 8 Departamento de Quimica Organica, Facultad de Optica y Optometria, Complutense University of Madrid, Madrid, Spain; 9 Department of Biochemistry and Molecular Biology, School of Chemistry, Complutense University of Madrid, Madrid, Spain; 10 Department of Environmental Health, Harvard T.H. Chan School of Public Health, Boston, MA USA

**Keywords:** Alarmins, allergic diseases, biologics, epithelial barrier, type 2 immunity, Immunology, Cytokines

## Abstract

Significant advancements have been made in understanding the cellular and molecular mechanisms of type 2 immunity in allergic diseases such as asthma, allergic rhinitis, chronic rhinosinusitis, eosinophilic esophagitis (EoE), food and drug allergies, and atopic dermatitis (AD). Type 2 immunity has evolved to protect against parasitic diseases and toxins, plays a role in the expulsion of parasites and larvae from inner tissues to the lumen and outside the body, maintains microbe-rich skin and mucosal epithelial barriers and counterbalances the type 1 immune response and its destructive effects. During the development of a type 2 immune response, an innate immune response initiates starting from epithelial cells and innate lymphoid cells (ILCs), including dendritic cells and macrophages, and translates to adaptive T and B-cell immunity, particularly IgE antibody production. Eosinophils, mast cells and basophils have effects on effector functions. Cytokines from ILC2s and CD4+ helper type 2 (Th2) cells, CD8 + T cells, and NK-T cells, along with myeloid cells, including IL-4, IL-5, IL-9, and IL-13, initiate and sustain allergic inflammation via T cell cells, eosinophils, and ILC2s; promote IgE class switching; and open the epithelial barrier. Epithelial cell activation, alarmin release and barrier dysfunction are key in the development of not only allergic diseases but also many other systemic diseases. Recent biologics targeting the pathways and effector functions of IL4/IL13, IL-5, and IgE have shown promising results for almost all ages, although some patients with severe allergic diseases do not respond to these therapies, highlighting the unmet need for a more detailed and personalized approach.

## Introduction

Allergic diseases represent a substantial burden on global health, with nearly one billion cases contributing significantly to morbidity, mortality, and healthcare costs. These diseases encompass a wide spectrum, including asthma, allergic rhinitis, chronic rhinosinusitis with nasal polyposis (CRSwNP), atopic dermatitis (AD), and food and drug allergies [1–3]. These clinically diverse diseases often present with a type 2 inflammatory signature in the majority of affected patients, although not universally. Specifically, a subset of patients demonstrates nontype 2 mechanisms, which may operate independently or in conjunction with type 2 pathways. Common pathophysiological features among patients with type 2 diseases include epithelial barrier dysfunction, cellular infiltration into tissues, tissue remodeling, microbiome alterations and immune dysregulation [1–3].

Our research on epithelial barriers started in 2000 with the demonstration of the mechanisms of type 2 diseases. Specifically, eczema, asthma and chronic rhinosinusitis with the death of epithelial cells lead to chronic epithelial barrier defects and periepithelial inflammation caused by innate and adaptive immune mechanisms [4–6]. The early understanding of epithelial barrier functions linked to type 2 diseases was “keeping away” from allergens, toxins, pollutants and microbes as the definition of barrier function. “Washing away” refers to the draining of inflammatory cells and cytokines by opening epithelial barriers and “suppressing” the functions of regulatory cytokines released by T cells and other cells on barrier surfaces during type 2 inflammation in healthy individuals exposed to high doses of allergens [7, 8]. Epithelial barrier defects have been demonstrated in asthma, AD and chronic rhinosinusitis for genetic reasons, epithelial barrier toxic substances and immune system cells and cytokines involved in the type 2 response, mainly IL-4 and IL-13 [9–12]. Certain epithelial barrier-damaging lifestyle-related or environmentally friendly substances, such as detergents, food emulsifiers, and air pollutants, have been demonstrated to cause epithelial barrier damage, alarmin release and tissue inflammation [13–23]. These works resulted in the development of the broad “epithelial barrier theory”, which posits that epithelial barrier dysfunction, induced by environmentally toxic substances linked to industrialization, urbanization, and modern life, results in the formation of a compromised epithelial barrier [1]. This barrier dysfunction coexists with microbial dysbiosis in the form of decreased commensals and colonization of opportunistic pathogens, bacterial translocation to the inter- and subepithelial areas, tissue and circulatory inflammation, and immune dysregulation. The combination of these diseases has been linked to the increasing prevalence of allergic, autoimmune, neuroimmune and other chronic diseases and their exacerbation [1, 24].

**Fig. 1:**
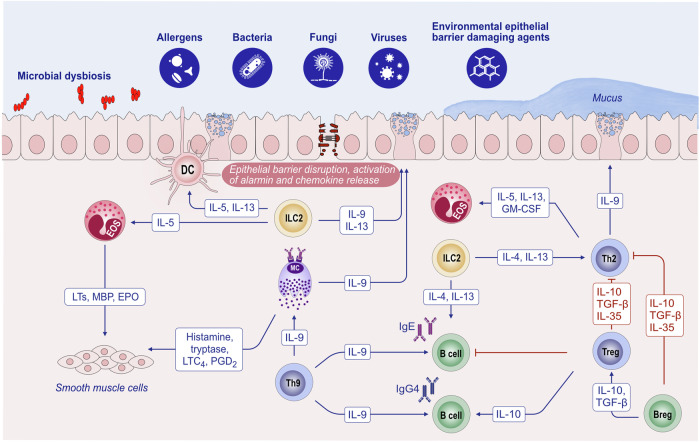
**Overview of the mechanisms of type 2 immune responses**. Epithelial barrier disruption during exposure to allergens, bacteria, fungi, viruses and environmental epithelial barrier-damaging agents and inflammation can lead to the opening of the epithelial barrier and allow the penetration of allergens through tissues. In addition, microbial dysbiosis occurs with the colocalization of opportunistic pathogens and the loss of commensals. Damaged epithelial cells release chemokines and alarmins, which activate innate lymphoid cells and dendritic cells. Matured DCs migrate to local lymph nodes and present processed allergen peptides to naive T cells through MHC class II molecules. Naive T cells in the presence of IL-4 differentiate into Th2 cells. The type 2 cytokines IL-4, IL-5, IL-9 and IL-13 are produced not only by Th2 cells but also by ILC2s. IL-4 and IL-13 are involved in IgE class switching in B cells. IgE binds to FcεRI on the surface of mast cells and sensitizes them. The subsequent release of mast cell-associated mediators, such as histamine, tryptase, prostaglandins, leukotrienes and cytokines, induces goblet cell hyperplasia, smooth muscle contraction, and increased vascular permeability. IL-5 induces eosinophilia. Immunoregulatory cytokines, such as IL-10, TGF-β, and IL-35, released by T regulatory (Treg) cells can suppress type 2 as well as Th1, Th9 and Th-17 responses. IL-10-producing Breg cells also inhibit effector T cells. DC dendritic cells, EOS eosinophil, EPO eosinophil peroxidase, GM-CSF granulocyte‒macrophage colony‒stimulating factor, IL interleukin, ILC innate lymphoid cells, LT leukotriene, LTC4 leukotriene C4, MBP major basic protein, MC mast cells, PGD2 prostaglandin D2, TGF-β transforming growth factor-β, TSLP thymic stromal lymphopoietin

Substantial research over the past decades has established the pivotal role of type 2 immunity and type 2 cytokines in allergic pathology (Fig. 1). Upon exposure to allergens, infectious agents, and environmentally toxic substances, skin and mucosal epithelial cells release alarmins, namely, thymic stromal lymphopoietin (TSLP), interleukin (IL)-25, and IL-33. These cytokines can directly induce type 2 cytokine production in group 2 innate lymphoid cells (ILC2s) and T helper 2 (Th2) cells [25, 26]. Alarmins are released together with many chemokines, attract type 2 response-related cells and sometimes activate inflammasomes and IL-1b [17, 21, 22, 27–29]. Like alarmins, cytokines involved in the type 2 response, including IL-4, IL-5, IL-9, IL-13, and IL-31, are also relevant in orchestrating one another and involve cells and overall type 2 immunity [3]. Naive CD4 + T cells differentiate into Th2 cells under the influence of IL-4, which also stimulates isotype class switching of B cells to produce immunoglobulin E (IgE) and increases the expression of the adhesion molecules VLA4 and VCAM-1 for the tissue migration of Th2 cells and eosinophils, opening epithelial barriers [30, 31]. IL-5 primarily promotes the maturation and recruitment of eosinophils. IL-9, secreted by Th9 cells, induces eosinophilic inflammation, mast cell (MC) growth, mucus hypersecretion, and airway hyperresponsiveness (AHR). IL-13 regulates IgE-producing B-cell proliferation, goblet cell hyperplasia, mucus hypersecretion and AHR and opens the epithelial tight junction (TJ) barrier [31, 32]. IL-31 initiates neuroimmune circuits, stimulating itch and neuronal outgrowth [33].

Type 2 inflammatory diseases exhibit shared mechanisms and therapeutic targets. In contrast to several decades ago, they were associated with multiple comorbidities that affect many organs at the same time as chronic inflammation, epithelial barrier damage and microbial dysbiosis [24]. This review delves into the intricate mechanisms of type 2 immunity, focusing on epithelial barrier dysfunction, the roles of cytokines and alarmins, and the complex cellular interactions involved. Additionally, it examines the implications of these mechanisms for therapeutic interventions, highlighting potential targets for treating type 2 immune-mediated diseases.

## Type 2 Immunity in Allergic Diseases

Type 2 immunity represents a very dedicated immune response to ameliorate the helminth burden in tissues. It does so by killing or expulsing them while simultaneously limiting tissue injury, maintaining tissue homeostasis and contributing to regeneration and fibrosis [31, 34–36]. In particular, the expulsion response against helminth larvae represents all the features of a full-blown type 2 immune response. A series of molecular events are exciting to ensure the cosurvival of the worm and the host. In 1932, Willem Löffler described eosinophilic pneumonia directed against ascaris, hookworms, Toxocara and Schistosoma [37, 38]. During their life cycle, infection occurs when fertilized eggs are ingested. The eggs hatch in the intestine, and the larvae migrate to portal veins and then pass through the vena cava inferior, right heart, and pulmonary artery and enter the lungs. The size of the larvae ranged between 0.5 and 1 mm. The growing larvae of the worms cause eosinophilic pneumonia with cough, as initially described by Löffler. Larvae must be fully expelled from the lungs before they become adults to accommodate their substantially large size of 15–20 cm, which can cause severe occlusions in the bronchial tree. There is no space in the lungs for adult worms to grow, which becomes a major threat to the survival of both the host and the parasite. Accordingly, the larvae are fully expulsed from the lungs when they are small and swallow where they find sufficient space in the gut to become adults. Similarly, an expulsion-like pathophysiology also occurs as an immune response to skin parasites, such as scabies [39]. The main aim of the type 2 response here is to drain the danger away from deep tissues, resulting in severe itching, scratching, eosinophilia and transepidermal drainage of the inflammation through and out of the skin, similar to atopic dermatitis [39, 40].

### Type 2 immunity and asthma

Asthma is commonly classified as type 2 or nontype 2 on the basis of the expression levels of blood/sputum eosinophils, exhaled nitric oxide (FeNO), and serum IgE and the presence of relevant allergen-specific IgE [3, 41]. Eosinophilic asthma can be allergic asthma in the presence of allergen-specific IgE, skin test positivity and clinical allergic disease, or nonallergic eosinophilic asthma in the absence of specific IgE and clinical allergic disease [42]. Reversible airflow obstruction, chronic inflammation, and airway hyperresponsiveness and remodeling are the hallmarks of asthma, which manifests itself in a variety of phenotypes and endotypes with distinct pathophysiological mechanisms [43, 44]. Remodeling, a major pathogenetic factor in asthma, involves the injury and repair cycle that can occur in severe or prolonged chronic disease [45, 46]. Originally, remodeling was defined as the thickening of the airway basement membrane that restricts the airway lumen and airflow, which was suggested to result from ongoing airway inflammation in an epithelial barrier leaky state, with an effort to develop a second layer of the subepithelial tissue barrier. It also includes chronic airway inflammation, goblet cell hyperplasia, airway smooth muscle hypertrophy, and edema. These alterations result in persistent airflow obstruction that does not respond to bronchodilators or the anti-inflammatory effects of corticosteroids [47]. Persistent airflow obstruction leads to poor asthma control and a greater risk of exacerbations [48].

Airway epithelial cells serve as the primary defense barrier between the external environment and internal structures, protecting against pathogens, allergens, and chemical irritants. This defense involves the release of epithelial alarmins, such as IL-25, IL-33, and TSLP, in response to epithelial damage. Notably, house dust mites, one of the most clinically relevant perennial allergens responsible for asthma exacerbation, exaggerate rhinovirus-induced epithelial RIG-I inflammasome activation and mature IL-1β release, which subsequently leads to compromised dynamics of RIG-I-dependent type I/III IFN responses. This imbalanced RIG-I signaling results in less effective virus clearance and sustained inflammasome- and IFN-dependent airway inflammation in asthma [49].

**Fig. 2:**
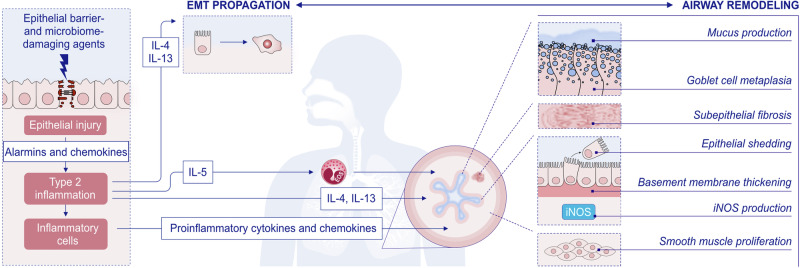
**Type 2 response and remodeling in the pathogenesis of asthma**. Exposure of the epithelial barrier and microbiome to damaging environmental agents can lead to airway damage and induce alarmin production, followed by type 2 inflammation. Increased activation of the epithelium leads to signaling to migrating inflammatory cells and activation of resident tissue mesenchymal cells, such as smooth muscle cells and fibroblasts. IL-4 and IL-13 produced by Th2 cells and ILC2s lead to extracellular matrix propagation and airway remodeling. In addition, IL-5 recruits eosinophils to periepithelial tissues and leads to an eosinophilic response. Both stromal and inflammatory cells produce proinflammatory cytokines and chemokines. Progressive structural changes, including mucus production, goblet cell metaplasia, subepithelial fibrosis, epithelial shedding, basement membrane thickening, iNOS production and smooth muscle proliferation, may lead to airway remodeling. The proinflammatory environment generated by airway remodeling sustains the inflammatory response. EGF epidermal growth factor, EMT epithelial mesenchymal transition; EOS, eosinophils; FGF, fibroblast growth factor; IL, interleukin; PDGF, platelet-derived growth factor; TGF-β, transforming growth factor-β; VEGF, vascular endothelial growth factor

Single blockade of TSLP, IL-25, or IL-33, which stimulate ILC2 and Th2 cells to release type 2 cytokines [25, 50, 51], decreases airway inflammation and hyperresponsiveness in murine asthma models [25, 52], and concurrent blockade of all three cytokines produces more pronounced effects [53]. The receptors of these alarmins are found on various cells, including dendritic cells, eosinophils, basophils, MCs, ILC2s, and macrophages. Type 2 inflammation with IL-4 and IL-13 at its core creates several vicious cycles, such as continuation of epithelial barrier damage and allergen exposure to deeper tissues; growth, expansion and survival of various inflammatory cells; smooth muscle hyperplasia; mucus production; increased migration of inflammatory cells; and tissue eosinophilia. Epithelial damage initiates cyclical inflammation, remodeling, and the subsequent production of proinflammatory mediators, and these processes are maintained in chronic and severe disease (Fig. 2). Th2 cells and ILC2s, which generate type 2 cytokines such as IL-4, IL-5 and IL-13 along with other inflammatory mediators, are the main drivers of type 2 inflammation.

IL-4 and IL-13 play crucial roles in many aspects of airway changes in asthma, whereas IL-5 supports the development and amplification of eosinophilic inflammation and the induction of airway remodeling. IL-5 also promotes the growth of other type 2 cells, such as basophils and mast cells [2, 54]. Both IL-4 and IL-13 contribute to the generation of IgE and B-cell class switching, which in turn triggers the degranulation of mast cells and basophils and the subsequent release of proinflammatory mediators. IL-4 and IL-13 induce smooth muscle cell proliferation, hyperplasia and contractibility, epithelial shedding, goblet cell hyperplasia and mucus production and contribute to fibrosis and reticular base membrane thickening [55, 56]. IL-4 and IL-13 further contribute to bronchial epithelial dysfunction by decreasing the expression of intercellular tight junction proteins, such as claudin 18.1, and increasing the expression of histone deacetylases 1 and 9 [2]. Stimulation of goblet cell hyperplasia and subsequent excessive mucus production are other effects of IL-4 and IL-13, which is of particular interest and importance: IL-13Rα2 has a potential role in IL-13-induced MUC5AC and ciliary changes through the ERK1/2 signaling pathway in the nasal epithelium. IL-13Rα2 may contribute to airway inflammation and aberrant remodeling, which are the main pathological features of CRSwNP [57]. Finally, by affecting the expression of vascular adhesion molecules (VCAMs), for example, VCAM-1, eosinophil recruitment to the airway occurs via eosinophilic inflammation and eventual remodeling.

Remodeling is further intensified by other inflammatory cells, such as MCs and eosinophils, which release mediators such as transforming growth factor-β (TGF-β), cationic proteins, and cytokines, thereby promoting fibrosis [58, 59]. TGF-β functions as a key mediator of airway remodeling by inducing epithelial‒mesenchymal transition. In addition to TGF-β, numerous cytokines, such as platelet-derived growth factor (PDGF), fibroblast growth factor (FGF), epidermal growth factor (EGF), vascular endothelial growth factor (VEGF), and chemokines (e.g., CXCL2, CXCL3, and IL-8/CXCL8), also contribute to airway remodeling in asthma, either directly or indirectly [60]. Remodeling is a complex process involving many molecules that act as either agonists or antagonists of the type 2 immune response. For example, LIGHT, a tumor necrosis factor (TNF) family member, acts together with TGF-β to promote airway remodeling [61].

### Type 2 immunity and atopic dermatitis

**Fig. 3:**
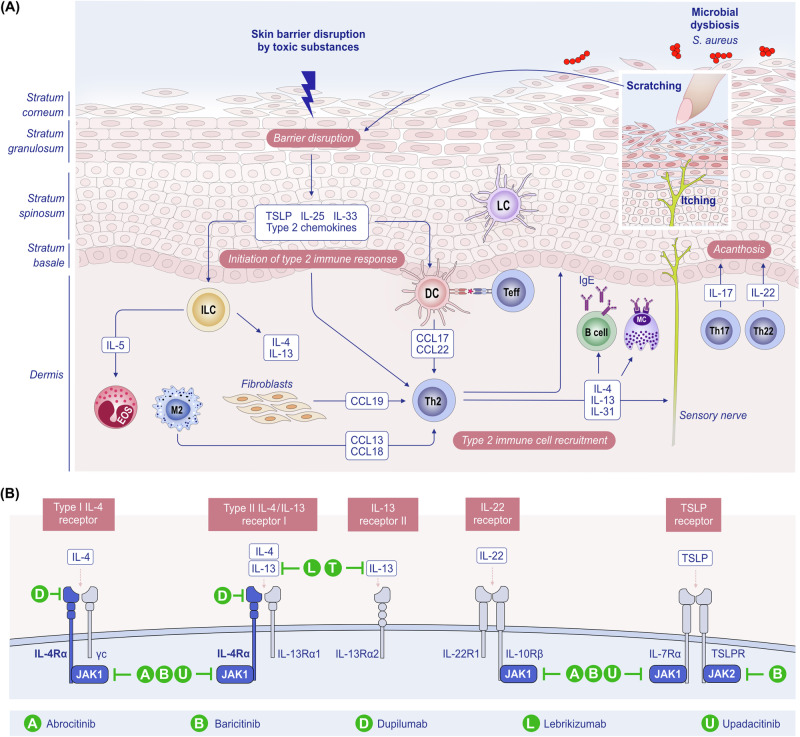
**Type 2 response in AD**. **A** Skin barrier disruption by toxic substances leads to the upregulation of alarmins such as TSLP, IL-25, IL-33 and type 2 chemokines. These alarmins activate ILC2s and Th2 cells, triggering type 2 inflammation. Additionally, the activation of DCs, M2 macrophages, and fibroblasts results in the production of type 2 chemokines, which attract Th2 helper T cells to the lesion. This cascade contributes to further barrier dysfunction, mast cell activation, IgE production by B cells, and direct activation of sensory nerves, causing itch. The following itch-scratch cycle exacerbates barrier dysfunction, initiating a vicious cycle of inflammation and barrier disruption in AD skin. **B** Description of receptors for IL-4, IL-13, IL-22, TSLP, and Janus kinases alongside biologics that have been approved for the treatment of AD. AD atopic dermatitis, DC dendritic cell; Ig immunoglobulin, IL interleukin, ILC2 type 2 innate lymphoid cell, JAK Janus kinase, TSLP thymic stromal lymphopoietin

AD is classically recognized as a Th2–skewed inflammatory disease [62–64]. The skin consists of various cell populations that cooperatively maintain homeostasis (Fig. 3A). Technical advancements, particularly single-cell transcriptomics, have revealed the complexity of the immune pathogenesis of AD [62, 63, 65–67]. Together with the significant immune shift to Th2 cell abundance in AD patients, Th2-specific DCs display signatures associated with skin-homing factors (ITGA4, ITGB1, and C-C chemokine receptor (CCR) 2), with low CD103 (ITGAE) and high CD11c (ITGAX) levels [67]. Several AD-specific activated immune cells—such as CCL13- and CCL18-expressing macrophages, CCR7-expressing DCs and T cells, and COL6A5- and C-C motif ligand (CCL) 19-expressing fibroblasts—have been identified in lesional AD skin [62, 66]. IL4R-, FCER2-, and IgG-expressing memory B cells are increased in AD, and these B cells are associated with atopic diseases via IgE production [68]. Upregulation of the nuclear factor-kappa B pathway may result in chronic dermatitis in AD skin. Inhibitory kappa B kinase (Ikk)β deletion leads to increased expression of CCL11 by fibroblasts, which induces eosinophilia and shifts the inflammatory response toward a type 2 immune response [69]. Ikkβ-deficient fibroblasts in facial skin produce phenotypes similar to those observed in AD, including scratching behaviors [70, 71]. Chronic nodular prurigo (CNPG) is also a type 2 inflammatory skin disease characterized by a chronic itch-scratch cycle. Recently, IL24+ and CXCL14^low^ CNPG-specific fibroblasts were reported to be distinct from AD fibroblasts [72]. It has been reported that IL-13 induces IL-24 secretion within the extracellular matrix. IL-24 downregulates filaggrin expression in keratinocytes via STAT3 and thereby promotes barrier dysfunction [73]. The well-known itch inducers IL31 and oncostatin M are increased in AD and CNPG patients; in addition, increased levels of neuromedin B in the fibroblasts of CNPG patients compared with those in AD patients and HCs have been identified. It is known that type 2 cytokines themselves also induce itch [74]. It has been reported that heterogeneous systems of itch underlie AD. Wang et al reported that basophils promote a mast cell-independent form of IgE-mediated itch. Allergen-stimulated basophils produce leukotriene C4 and activate sensory nerves. This leukotriene C4-CysLTR2 neuronal signaling axis mediates acute itch flares in AD [75].

The TSLP influences T-cell activity, selective white adipose tissue loss, sebum secretion, and sebum-associated antimicrobial peptide expression in human skin homeostasis [76]. In addition, TSLP recruits Th2-cell clusters that produce IL-4 and IL-13. These type 2 cytokines can also promote sebum secretion and regulate the skin barrier [77]. Obesity further complicates the type 2 immune response. The activity of the nuclear receptor peroxisome proliferator activated receptor-γ (PPARγ) in Th2 cells from obese mice is decreased, and obesity converts a classically Th2-driven inflammatory skin response to more severe Th17-driven dermatitis [78]. Interestingly, the treatment of obese mice with a small-molecule PPARγ agonist has been shown to limit the development of Th17 pathology and unlock the therapeutic responsiveness of the treatment target to Th2 inflammation.

Biologics that antagonize type 2 immune responses, such as IL-4 receptor subunit α (IL4R α) inhibitors, are effective in treating allergic diseases such as AD, asthma, EoE, and chronic rhinosinusitis with nasal polyps. Owing to the pathogenesis of AD, 20 to 50% of patients experience no substantial improvement with existing therapies. Recent advances in anti-type 2 biologic drug development include the approval of biologics showing good efficacy in AD, such as dupilumab (anti-IL4R α) in 2017, tralokinumab (anti-IL-13) in 2021, lebrikizumab (anti-IL-13) in 2023, nemolizmab (anti-IL-31) in 2024, oral Janus kinase (JAK) inhibitors (JAKi) targeting JAK1/2 (baricitinib) in 2020, JAK1 (upadacitinib) and JAK1/2 (ruxolitinib) in 2021, and JAK1 (abrocitinib) in 2022 [79–82] (Fig. 3B, Table 1). JAK and signal transducer and activator of transcription (JAK–STAT) signaling pathways mediate the effects of central cytokines (e.g., IL-4, -5, -13, -22, -31, and TSLP) in the pathogenesis of AD. A high proportion of nonresponders to dupilumab (47.2% with 200 mg abrocitinib; 35.2% with 100 mg abrocitinib) respond to abrocitinib 12 weeks after therapy is switched [83].

**Table 1:** FDA-approved therapeutics targeting type 2 immune pathways in allergic diseases

Drug	Target	Indication	Year of FDA approval (if applicable)	Mechanism of action
**Omalizumab**	Anti-IgE	Severe allergic asthma, chronic urticaria, CRSwNP, Food allergy	Asthma: 2003, Urticaria: 2014, CRSwNP (EU) FA: 2024	Prevents IgE binding to FcεRI, reducing mast cell activation
**Mepolizumab**	Anti-IL-5	Severe eosinophilic asthma, CRSwNP, EGPA	Asthma: 2015, CRSwNP: 2021	Inhibits IL-5, reducing eosinophil production and survival
**Reslizumab**	Anti-IL-5	Severe eosinophilic asthma	2016	Inhibits IL-5 activity, lowering eosinophil counts
**Benralizumab**	Anti-IL-5Rα	Severe eosinophilic asthma	2017	Induces eosinophil apoptosis via ADCC
**Dupilumab**	Anti-IL-4Rα	Moderate-to-severe AD, Asthma, CRSwNP, EoE prurigo nodularis, COPD	AD: 2017, Asthma: 2018, CRSwNP: 2019, EoE: 2022 PN: 2024, COPD: 2024	Inhibits IL-4R signaling, reduces type 2 inflammation
**Tezepelumab**	Anti-TSLP	Severe asthma	2021	Blocks TSLP signaling, reducing airway inflammation
**Tralokinumab**	Anti-IL-13	AD	2021 (EMA/FDA)	Reduces IL-13-mediated inflammation in AD
**Lebrikizumab**	Anti-IL-13	AD	2023 (FDA)	Reduces IL-13 signaling, improves AD lesions
**Baricitinib**	JAK1/JAK2 inhibitor	AD	2020	JAK1/2 inhibitor
**Upadacitinib**	JAK1 inhibitor	AD	2021	ATP competitive JAK1/2/3 inhibitor(JAK1 strongest)
**Abrocitinib**	JAK1 inhibitor	AD	2022	JAK1 inhibitor
**Ruxolitinib**	JAK1/JAK2 inhibitor	AD (USA)	2021	JAK1/JAK2 inhibitor
**Nemolizmab**	Anti-IL-31	Prurigo nodularis	2024	Block IL-31 signaling, reducing itch

*AD* atopic dermatitis, *ADCC* antibody-dependent cellular cytotoxicity, *COPD* chronic obstructive pulmonary disease, *CRSwNP* chronic rhinosinusitis with nasal polyposis, *EGPA* eosinophilic granulomatosis with polyangiitis, *EoE* eosinophilic esophagitis, *JAK* Janus kinase, *TSLP* thymic stromal lymphopoietin

Recent research on noninvasive methods to predict AD has shown that free sphingoid bases of different chain lengths and CCL17 (TARC) in tape strip samples are altered in infants who later developed AD [84, 85]. It has been reported that RNA-seq data from tape strips can identify differential gene expression between AD and non-AD samples [86, 87]. Common inherited loss of filaggrin, significant reduction in natural moisturizing factor, and water content are strongly associated with AD onset by the age of 4 weeks [88]. AD is characterized by a defective skin barrier—a potential mechanism underlying the development of allergic comorbidities in early life in children with AD. Progressive longitudinal accumulation of NKG2D^low^ CD56^dim^ NK cells in allergen-sensitized children has been reported. Importantly, an increased number of these NK cells is positively associated with skin barrier function, as assessed by transepidermal water loss (TEWL) [89]. Tape strip-induced barrier impairment in mouse skin triggers antigen-driven allergic skin inflammation [90]. Electrical impedance spectroscopy (EIS) has recently been described as a useful tool for detecting epidermal barrier function. The reduction in EIS represents epithelial barrier damage in vivo and in ex vivo human skin [23, 91, 92]. The effect of detergent exposure on skin barrier impairment can be demonstrated in a few seconds by using this method [23]. Compared with that of healthy controls, the skin of AD patients has a lower EIS value, which is restored to normal values after treatment [91]. In addition, EIS can detect skin barrier dysfunction and differentiate the skin of children with AD from healthy skin, suggesting that EIS may be a potential tool for predicting future AD development [93].

### Type 2 immunity and chronic rhinosinusitis

CRS is a heterogeneous disease characterized by differences in inflammation in the upper airways. Traditionally, CRS is categorized into two primary phenotypes: CRS with nasal polyps (CRSwNP) and CRS without nasal polyps (CRSsNP) [94]. There is a specific clinical phenotype named refractory sinusitis, which is defined as not responding to conventional functional endoscopic sinus surgery followed by oral or topical corticosteroids and antibiotics and is predominant with type 2 inflammation [95]. Recently, endotypes have been distinguished by underlying pathogenetic mechanisms and can be identified into three endotypes on the basis of the elevation of specific lymphocyte cytokines: type 1, which is characterized mainly by the Th1 cytokine IFN-γ; type 2, which is characterized by the Th2 cytokines IL-4, IL-5, and IL-13; and type 3, which is characterized by Th17 cytokines, including IL-17 [96]. Type 2 immune inflammation plays a significant role in the pathogenesis of chronic rhinosinusitis. When upper airway epithelial cells are exposed to external stimuli, including allergens, pathogens and chemicals, they are activated and release IL-25, IL-33 and TSLP. These cytokines lead to the activation of Th2 cells, ILC2s and dendritic cells, resulting in the overproduction of IL-4, IL-5 and IL-13 [97]. To better understand the mechanisms of type 2 inflammation in CRS, distinguishing Th2 cells is crucial. These cytokines (IL-4, IL-5 and IL-13) are markers for discriminating Th2 cells, and there are many other specific markers on the surface of Th2 cells, including CCR3, CCR4, CCR8, CXCR4, and ST2/IL-1 R4 [98, 99].

Epithelial barrier damage is also an important pathological feature of CRS. The physical barrier, mucociliary escalator, and local microbiome form the intact epithelial barrier [100]. The main structures of the physical barrier are tight junctions (e.g., ZO-1, occludin, and claudins as well as junctional adhesion molecule 1 proteins) and adherens junctions (e.g., the transmembrane proteins E-cadherin and nectin and the intracellular proteins α-catenin and β-catenin) [101]. Soyka et al. reported a defective epithelial barrier in patients with CRSwNP, along with reduced expression of TJ proteins linked to IFN-γ and IL-4 as effector cytokines [102]. Subepithelial fibrosis is also observed in CRS. Differentiated epithelial cells lose their characteristic shape, polarity, and intercellular junctions and then begin to proliferate and transform into a spindle-shaped fibroblast-like morphology with migratory capabilities [103]. IL-13 and IL-4 could be inducers of this process in CRS, which mainly activate myofibroblasts to promote ECM accumulation [102, 104].

Nasal mucus biomarkers have also been explored to distinguish T2 and non-T2 inflammation as noninvasive predictors. The high activity of eosinophils, mast cells, and basophil microparticles in nasal lavage fluid facilitates the identification of severe eosinophilic CRS [105, 106]. The cystatin SN, encoded by CST1, has strong prognostic and predictive value in the medical management of CRSwNP and increases eosinophil activation and IL-5 infiltration [107, 108]. The expression of the bone morphogenetic proteins (BMPs) BMP-2 and BMP-7 was identified as a vital predictor of recurrent CRSwNP [109]. Tissue plasminogen activator has been identified as a negative biomarker for T2 immune responses in nasal polyps, and inactivation of tissue plasminogen activator leads to excessive fibrin deposition [110]. B cells are pivotal sources of upregulated polyclonal, functional IgE in type 2 inflammation in CRSwNP. The class switch from recombination to IgE occurs in the presence of IL-4 in T2 CRSwNP [111]. Additionally, the deposition of eosinophil extracellular traps and Charcot–Leyden crystals in CRSsNP also underlies type 2 inflammation [111].

Since 2006, biological therapy with monoclonal antibodies (mAbs) used for the treatment of severe allergic asthma has also been shown to be effective for treating CRSwNP [112]. The type 2 cytokines IL-4, IL-5, and IL-13, as well as IgE and eosinophils, play essential roles in sustaining inflammation and promoting the formation of nasal polyps. Recent advances in mAbs indicate that currently available mAbs targeting eosinophilic or type 2 inflammation are available for the treatment of CRSwNP and provide significant improvements in severe and uncontrolled CRSwNP patients, as demonstrated in several high-quality phase I‒III randomized controlled clinical trials, such as those for dupilumab, omalizumab, and mepolizumab (Table 1) [113, 114]. Dupilumab, which is directed against the IL-4 receptor alpha, is the first biologic therapy approved in the European Union and the USA for the treatment of uncontrolled CRSwNP [115]. Omalizumab, an anti-IgE therapy, is the second biologic therapy approved in the European Union. Mepolizumab, which inhibits interleukin IL-5 immune responses, is the third biologic approved for CRSwNP [116, 117]. Furthermore, several clinical trials on type 2 biologics, such as CM310 (anti-IL-4Rα), reslizumab (anti-IL-5), and benralizumab (anti-IL-5Rα), have also shown dramatic improvements in both clinical and patient-reported outcomes (Table 1) [118, 119].

### Type 2 immunity and eosinophilic esophagitis

EoE is a chronic, immune-mediated disease characterized by inflammation primarily centered in the esophageal mucosa. A key diagnostic criterion for EoE is the presence of more than 15 eosinophils per high-power field. The disease prominently features a Th2 immune response. EoE strongly correlates with allergies, not only because it is often accompanied by atopic conditions but also because food and aeroallergens can trigger symptoms [120–122]. Epithelial barrier dysfunction in the esophagus may contribute to the onset or progression of the disease by triggering a Th2 response and allowing allergens to infiltrate deeper tissues, further stimulating the inflammatory milieu.

The damaged epithelial barrier secretes the alarmins TSLP, IL-25, and IL-33, which induce the maturation of T helper cells and ILC2s. These cells, in turn, produce IL-4, IL-5, IL-9, and IL-13, as well as TGF-β and eotaxin [123, 124]. Eosinophils are then recruited to the esophagus, largely by IL-5, which is overexpressed in EoE, along with T cells and mast cells [123, 124]. Genetic studies have revealed that epithelial-derived genes such as calpain 14 and TSLP are dysregulated in EoE. This dysregulation leads to an impaired barrier, partly due to the loss of desmoglein 1 expression [125]. These genes are also associated with the type 2 cytokine gene IL-4 and are induced by IL-13 [126].

Like patients with other atopic conditions, EoE patients often show IgE sensitization to aero- and food allergens. However, treatment with omalizumab, which targets this immunoglobulin, has not been successful, indicating that EoE is not driven by an IgE-mediated mechanism [127, 128]. Recent evidence suggests that IgG4, which is specific to food allergens, might play a role in disease pathophysiology [127, 129]. This ‘modified type II response’, which promotes IgG4 production while suppressing IgE responses, potentially explains the low levels of IgE in EoE and why anti-IgE treatments are unsuccessful [130]. Other therapeutic agents targeting components of the Th2 response have shown efficacy in reducing inflammation in EoE. IL-5-directed agents, namely, mepolizumab, reslizumab, and benralizumab, have been shown to reduce esophageal eosinophilia but not EoE symptoms (Table 1) [131]. Likewise, biologics against the IL-4 and IL-13 signaling pathways have failed to improve symptoms, although they successfully treat eosinophilia [132, 133]. However, an IL-4R-targeted treatment that is already in use for other atopic conditions, dupilumab, was recently approved by the Food and Drug Administration for the treatment of EoE, resulting in both histologic remission and symptom improvement (Table 1) [134].

### Type 2 immunity and food allergy

Food allergies are defined as adverse reactions to food via immunological mechanisms and are usually divided into IgE-mediated, non-IgE-mediated and mixed types, the latter including both IgE-mediated and non-IgE-mediated mechanisms [135]. Regardless of type, recent advances have shown that the Th2-related response plays a central role in food allergies [136]. Sensitization to food allergens can occur in the gastrointestinal tract, skin, and oral cavity but rarely in the respiratory system [137].

After food intake, further processing takes place in the gastrointestinal system via various enzymes and gastric acid. The antigens from food then pass through epithelial cells and specialized M cells, which are located above Peyer’s patches [136]. Passage through M cells leads to IgA induction, which serves to neutralize the antigen [138]. On the other hand, the transport of soluble antigens can occur transcellularly in vesicles or paracellularly between cells, whereby the latter is restricted by the tight junctions between enterocytes [139]. In addition to the epithelium, antigen uptake from the lumen can also occur directly by macrophages and dendritic cells, which are located between enterocytes with the help of their dendrites [140]. After the antigen is transported by CX3CR1+ macrophages to CD103+ dendritic cells, these cells migrate to mesenteric lymph nodes and present the antigen to naive T cells [140]. CX3CR1+ dendritic cells tend to induce inflammation, whereas CX3CR1- dendritic cells have tolerogenic properties by promoting the development of Tregs in mesenteric lymph nodes [141]. The expression of OX40 ligand (OX40L) on dendritic cells also contributes to Th2 cell differentiation [137]. Regulatory T cells play a central role in the induction of oral tolerance through inhibitory cytokines such as IL-10 and TGF-ß and suppressive signaling through programmed cell death protein 1 (PD-1) and cytotoxic T lymphocyte-associated protein 4 (CTLA4) [142]. IL-10-producing B cells have also been shown to increase IgG4 levels and reduce IgE production [141, 143]. The transition from tolerance to allergy depends on the nature of the immune response to food antigens; first, these antigens must cross the intestinal barrier, and then Th2-promoting signals are required to initiate allergic sensitization [136]. The nature of antigens and the integrity of the epithelial barrier are key in these first steps [137]. One example is the binding of glycans present on allergenic peanut proteins to the C-type lectin receptors (CLRs) of dendritic cells, which activate DCs to promote the Th2 response [144]. Binding to the CLR dendritic cell-specific intercellular adhesion molecule-3-grabbing nonintegrin on human monocyte-derived DCs (moDCs) has also been demonstrated with various food allergens, including hazelnut, walnut and egg white [145]. Exposure to hazardous molecules from the environment and food processing also contributes to damage to the epithelial barrier, alteration of the gut microbiome and subepithelial inflammation, which may lead to a Th2-type response [146]. The alarmins released from the damaged epithelial barrier, such as IL-25, IL-33, and TSLP, contribute to the Th2 response [147].

The absence of strong Th1 signaling and the presence of IL-4 can lead to Th2 differentiation. In addition to activated naive T cells, mast cells, basophils and ILC2s may be sources of IL-4 [137]. Another Th2 subset named Th2A cells was shown to drive IgE class switching in allergic patients [148]. The cytokines released by Th2 cells, especially IL-4 and IL-13, induce the switch of the B-cell class from IgG to IgE. Follicular T helper (Tfh) cells, which secrete IL-4, and the Tfh13 subtype, which secretes IL-4, IL-5 and IL-13, are associated with high-affinity IgE, which is involved in anaphylaxis with food allergens [149]. The formation of IgE from B cells to food antigens leads to the first step of sensitization. This food-specific IgE binds to the FcεRI receptors on mast cells and basophils. Re-exposure to this food antigen leads to cross-linking of the allergen with the receptors and further release of preformed mediators from mast cells and basophils into the bloodstream. These mediators, including histamine, tryptase, leukotrienes, prostaglandins and platelet-activating factor, are responsible for the classic symptoms of IgE-mediated food allergies ranging from urticaria to anaphylaxis [150].

Food protein-induced enterocolitis syndrome (FPIES), a non-IgE-mediated immune reaction to food, is a myriad of clinical symptoms resulting from immune-mediated adverse reactions to food leading to repetitive vomiting to exhaustion within 1–4 hours of trigger consumption [151]. As the diagnosis of FPIES is clinical and does not require invasive testing, it remains difficult to elucidate the mechanisms behind the disease process, as it is now thought that local immune activation and neuroendocrine axis activation are responsible for the manifestations [152]. The attempts to create mouse models for FPIES have proven ineffective. Hence, disease modeling has been challenging. Once thought to be a type IV hypersensitivity reaction solely as a result of the type 1 immune response, emerging evidence has supported the role of the type 2 immune response in FPIES. Morita et al. reported that specific T-cell responses were skewed toward a Th2-orchestrated response in patients with non-IgE-mediated food allergies [153]. In another study by Wada et al., increased expression of CD69 was found on the surface of eosinophils, suggesting activation [154]. Immune cell–nerve interactions are well documented in peripheral tissues, suggesting bidirectional cross-talk. ILC2s express VIPR2, which, upon activation, leads to the secretion of IL-5, resulting in a positive feedback loop by interacting with the neural terminal and leading to further VIP secretion in the lung [155]. These findings suggest a complicated and multisystem relationship between the immune response, both type 1 and type 2, and the nervous system in the pathogenesis of FPIES. Why the same substances produce an IgE-mediated response in some patients and non-IgE-mediated responses in some patients are currently unknown.

Food allergies in patients with atopic dermatitis can manifest as early, IgE-mediated reactions such as urticaria and anaphylaxis as well as late non-IgE-mediated exacerbations of atopic dermatitis. Although atopic dermatitis itself is not an adverse reaction to food, the bidirectional relationship between atopic dermatitis and adverse reactions to food is well established. As mentioned earlier, disruption of the skin epithelial barrier leads to the initiation of a cascade of events leading to a type 2 immune response. These immune responses are not limited to only local tissues but also result in a systemic skew toward a type 2 immune response and the circulation of activated immune cells [1]. The passage of food allergens through leaky barriers leads to the encounter of dendritic cells and food allergens in a highly inflammatory milieu dominated by type 2 cytokines and immune cells. These signals may lead to the migration of dendritic cells to regional lymph nodes after the engulfment of allergens, resulting in sensitization. Allergen-specific T cells home to the intestine where they reside, leading to disease manifestations [156]. The sensitization of T cells to food allergens in the gut can result in the expansion of skin-homing allergen-specific T cells, exacerbating atopic dermatitis [157]. To conclude, the complex interplay between atopic dermatitis and food allergies involves both local and systemic immune responses, where skin barrier disruptions and food allergen exposure lead to sensitization and exacerbation of atopic dermatitis through a type 2 immune response.

## Cells with a Type 2 Response

### T helper 2 cells in the type 2 response

**Fig. 4:**
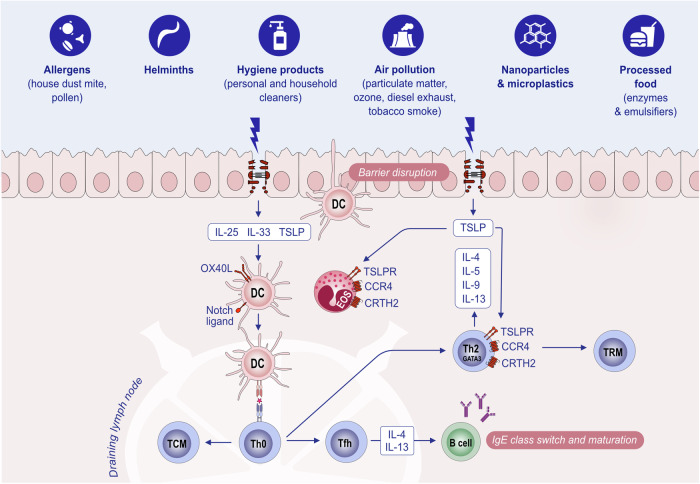
**Initiation of the type 2 immune response**. Exposure to allergens, helminths or epithelial barrier-damaging toxic substances causes epithelial alarmin release (TSLP, IL-25, and IL-33) and epithelial barrier impairment. Allarmins activate DCs, and activated DCs increase their OX40L or Notch receptor ligands and migrate to the draining lymph node, where they present antigens/allergens to naive T cells and activate these T cells to become Th2, Tfh and TCM cells. Th2 cells migrate to epithelial tissue and release type 2 cytokines such as IL-4, IL-5, IL-9, and IL-13. Epithelial alarmins further activate Th2 cells to produce more cytokines and cause an increase in TSLPR in Th2 cells. Tfh cells in the lymph node help IgE class switching and affinity maturation in B cells. Ig immunoglobulin, IL interleukin. OX40L ligand for OX40. TCM central memory T cell, Tfh T follicular helper cells, Th2 T helper 2 cells, TRM tissue-resident memory T cells, TSLP thymic stromal lymphopoietin

Th2 cells orchestrate type 2 responses in both health and disease. Th2 cells are characterized by the secretion of type 2 cytokines such as IL-4, IL-5, IL-9, IL-10, and IL-13; the transcription factor GATA-3; and cell surface receptors such as CCR4 and CRTH2 [158, 159]. Th2 polarization depends on costimulatory molecules and cytokines during the antigen presentation process by DCs in the draining lymph node. DCs in mucosal areas can be stimulated by epithelial alarmins to prime the Th2 response [160] (Fig. 4). Epithelial alarmin-conditioned DCs produce lower levels of IL-12, a major type 1 cytokine, and increase the expression of strong Th2-polarizing molecules such as OX40L or Notch receptor ligands. In turn, T cells primed with epithelial alarmin-conditioned DCs produce IL-4, IL-5, IL-13, and TNF-α [161, 162]. In addition, several types of DCs, such as CD301b+ DCs in the skin, have the intrinsic ability to prime Th2 responses [158, 163, 164]. The direct stimulation of Th2 cells with TSLP, which expresses higher levels of TSLPR than Th1 and Th17 cells do, induces type 2 cytokine secretion [158].

After Th2 cell priming by DCs, various subsets of Th2 cells can develop [165]. Effector Th2 (Teff) cells migrate back to tissues to orchestrate local tissue responses. Central memory T (TCM) and tissue-resident memory T (TRM) cells contribute to initiating, propagating, and sustaining type 2 immune responses in response to persistent antigen exposure. Additionally, DCs working together with B cells induce the generation of type 2 follicular helper T (Tfh) cells, which produce IL-4 and IL-13. IL-4 plays a crucial role in promoting IgE class-switching within B cells, whereas IL-13 is necessary for affinity maturation of IgE [166].

Following complete activation via T-cell receptor engagement and costimulation, naive CD4^+^ T cells undergo a radical shift in metabolism to support their proliferation, differentiation, and function [167]. This metabolic reprogramming not only facilitates the generation of energy and metabolites enabling catabolic and anabolic pathways [167, 168] but is also tailored to the type of CD4^+^ T helper cell subset desired for a particular response [169]. The differentiation and function of CD4^+^ T cells are influenced by glucose, amino acid and fatty acid metabolism [169]. Efficient polyamine metabolism is critical for accurate polarization of CD4^+^ T cells into specific subsets [170]. In particular, differentiation into Th2 cells, in terms of metabolism, involves the upregulation of GLUT1 upon activation [171] to facilitate glycolysis via a mammalian target of rapamycin (mTOR)-dependent mechanism [168, 171, 172]. Both components of functional mTOR, namely, mTOR complex 1 (mTORC1) and 2 (mTORC2), are essential for Th2 cell differentiation and function since their dysregulation greatly limits these processes [168, 171]. Similarly, fatty acid synthesis (FAS) is also important in the generation and function of effector Th2 cells since it supplies membrane components for freshly divided cells, among other roles [171]. Remarkably, although a reduction in FAS potentially enables the generation of resident memory Th2 cells, as was observed upon pharmacological inhibition of the FAS rate limiting enzyme acetyl-CoA-carboxylase 1 (ACC1) in mice [173], these cells are enriched in lipid metabolism-related gene expression in comparison to circulating memory Th2 cells [171]. Downstream of mTORC1 signaling, the lipid gene regulating the transcription factor peroxisome proliferator activated receptor-γ (PPAR-γ) not only regulates the uptake of lipids in activated naive CD4^+^ T cells but also facilitates resident memory Th2 cell function during recall responses, as observed in PPAR-γ knockout mouse models [171]. PPAR-γ has been reported to be elevated in the Th2 cells of individuals with asthma and allergies, and their assessment revealed, as expected, increased expression of lipid metabolism genes [171]. Collectively, these observations highlight metabolic differences within Th2 cell subsets throughout their lifespan, ranging from effector cells to resident memory cells, and call for more nuanced analysis in future studies.

### Type 2 CD8 + T cells (Tc2 cells) in the type 2 response

Although Th2 cells have been well established as central mediators of type 2 inflammatory responses, emerging evidence suggests that type 2 cytotoxic T (Tc2) cells also play a significant role in pathogenesis, influence disease severity and affect treatment outcomes [174]. Tc2 cells that produce Th2-like cytokines have been identified in airway and intraepithelial tissues [175]. When naive CD8 + T cells are cultured with IL-4 in vitro, they differentiate into a subset that produces IL-4, IL-5 and IL-13, which are characteristic of Tc2 cells [176]. The molecular mechanisms underlying Tc2 cell differentiation closely parallel those governing Th2 cell development. Transcriptional regulators commonly associated with Th2 lineage commitment, notably STAT6 and GATA3, are also pivotal in guiding Tc2 cell differentiation and promoting their type 2 cytokine profile [177]. Under the influence of IL-4, these factors reprogram the CD8 + T-cell cytokine profile, enabling the production of IL-4, IL-5, IL-13, and, in some cases, IL-9 and IL-10 instead of IFN-γ. In contrast to classical cytotoxic T lymphocytes (Tc1 cells), which are highly effective at killing target cells, such as virus-infected cells or tumor cells, Tc2 cells exhibit diminished cytotoxicity. Instead, they support type 2 immune responses by enhancing humoral immunity, recruiting eosinophils, and amplifying allergic inflammation [178–180]. Epigenetic modifications and metabolic reprogramming further stabilize the Tc2 phenotype, promoting its persistence in chronic inflammatory environments.

Within the lung, Tc2 cells are particularly abundant in severe eosinophilic asthma and during exacerbations [178]. Elevated IL-33 levels promote Tc2 cell expansion and maintenance, thereby skewing immune responses toward a more pronounced type 2 profile [181]. Mast cell–derived prostaglandin D2 (PGD2) and leukotriene E4 (LTE4) can activate Tc2 cells, prompting them to secrete type 2 cytokines [178, 182]. In contrast to Th2 cells, Tc2 cells exhibit reduced sensitivity to corticosteroids, implicating them in steroid-resistant forms of the disease [179, 183]. Furthermore, the mitochondrial enzyme Cyp11a1, which is involved in steroidogenesis, has been identified as a key regulator of Tc2 differentiation in steroid-refractory contexts [184]. Hypoxic conditions can also exacerbate Tc2 cell pathogenicity by increasing IL-13 production [185].

Within the context of allergic rhinitis, IL-4–producing CD8 + T cells help maintain ongoing type 2 inflammatory processes [186]. Moreover, therapies that promote immune tolerance, such as allergen immunotherapy, reduce the proportion of IL-4–secreting CD8 + T cells in patients experiencing intermittent allergic rhinitis [187, 188], suggesting that downregulation of Tc2 responses is integral to its therapeutic mechanism.

In AD, a similar pattern emerges. Individuals with AD display significantly greater frequencies of Tc2 cells than healthy controls do [189, 190]. While Tc2 cells constitute approximately 1% of CD8 + T cells in healthy individuals, their proportion can increase to approximately 4% in AD patients. Histamine, a critical mediator of allergic inflammation, enhances dendritic cell–mediated antigen cross-presentation and creates a milieu supportive of Tc2 cell accumulation [191, 192]. Blockade of the histamine H4 receptor reduces type 2 cytokine production and reduces the proliferation of both CD4+ and CD8+ cells in models of allergic contact dermatitis, underscoring the broader influence of histamine on Tc2-driven responses [193]. Single-cell RNA sequencing and proteomic investigations revealed that patients with AD receiving dupilumab still retain Tc2 cells as tissue-resident memory populations within the skin, whereas such cells are absent in individuals without the disease [194]. These observations suggest that Tc2 cells persist despite treatment and may contribute to disease relapse or suboptimal therapeutic responses.

### Eosinophils in type 2 response

Single-cell RNA-seq analysis of mouse eosinophils from different organs suggested distinctive subpopulations. These eosinophil subpopulations include eosinophil precursors and immature, circulating, basal, and active eosinophils across mouse tissues [195, 196]. Cumulative evidence suggests that eosinophils undergo extensive specialization in the intestines, driven by the microbiota [196].

The development, maturation, terminal differentiation, and release of eosinophils produced in the bone marrow from CD34+ progenitor cells are controlled by IL-5 and, to a lesser extent, by IL-3 and granulocyte‒macrophage colony‒stimulating factor (GM‒CSF) [197, 198]. In severe asthma, biological treatments that primarily target IL-5 have increased our understanding of the significant roles of eosinophils [199].

In the type 2 immune response, IL-5 activates eosinophils, and IL-9 recruits them along with mast cells. IL-4, IL-9, and IL-13 induce mucus production and enable B cells to produce IgE. These cytokines facilitate the migration of eosinophils and Th2 cells through the vascular endothelium. Notably, eosinophilia is enhanced through ILC2 activation by IL-33 and IL-25. This activation also contributes to general type 2 responses by releasing IL-5 and IL-13.

Eosinophils play crucial roles in allergic inflammation and airway tissue remodeling by releasing granule proteins, such as major basic protein (MBP) and eosinophil peroxidase (EPO), which can profoundly affect airway tissues when activated. These proteins also initiate airway damage [198]. The migration of eosinophils into airways marks the onset of inflammation in these tissues, thus influencing asthma severity. Several type 2 inflammatory cytokines facilitate the complex trafficking of eosinophils [200]. In this process, the adhesion of eosinophils to blood vessels via VCAMs is a critical step. The principal protein involved is VCAM-1, and its expression is regulated by IL-4. Through the binding of VLA-4 to VCAM-1, eosinophils are dispensed from blood vessels and directed toward the lungs and airways [201–203].

Elevated eosinophil levels in peripheral blood (>150 cells/μL) and sputum (>2%) are indicative of a type 2 inflammation phenotype and increase the risk of asthma exacerbation. It is possible that changes in TLR-7 function and IFN-γ production contribute to this condition [204]. Eosinophil activation, which primarily occurs in the late stages of the allergic response, is a crucial element of the type 2 inflammatory cascade and plays a central role in the pathogenesis of asthma. This activation involves the release of cytoplasmic granule mediators and the production of cytokines (mainly IL-1β, IL-6, IL-8, and IL-4), lipid mediators, and oxygen radicals. Activated eosinophils cause DC chemotactic activity, endothelial cell damage, inhibition of muscarinic receptors, disruptions in repair processes, and fibrosis induction, all of which lead to airway hyperactivity and remodeling [205–207]. There is a significant correlation between these mechanisms and the exacerbation of asthma.

### Dendritic cells and macrophages in the type 2 response

To orchestrate type 2 immune response initiation and maintenance, innate and adaptive immunity need to be coordinated through their respective cell subsets locally and systemically at the time of antigen/allergen presentation [208]. In humans, DCs, macrophages, and B cells are classified as professional antigen-presenting cells (APCs) [209].

DCs are defined as tissue-resident and circulating cells that perceive microbes and trigger innate immune reactions. Most DCs are spread in lymphoid tissues, the mucosal epithelium, and the organ parenchyma. Owing to their wide distribution, DCs are sentinels of infection that initiate a rapid immune response but also bridge innate to adaptive immunity [210].

In blood circulation in humans, DCs are classified on the basis of their expression of specific surface markers: (1) plasmacytoid DCs (pDCs) and (2) myeloid DCs (mDCs). After being activated, pDCs resemble plasma cells morphologically. They produce the antiviral cytokine type I interferon in response to viruses and may capture blood-borne microbes and carry their antigens for presentation to the spleen. mDCs can even be categorized into type 1 and type 2 mDCs (DC1 and DC2, respectively) [211]. In both humans and mice, DC2s constitute the main cell subset involved in the induction and expansion of Th2 cells in the airways, gut, and skin [212–215]. A recent study in a human model of asthma exacerbation revealed that local DC2–Th2 crosstalk may establish Th2 residence in the airway, license the pathogenic Th2 phenotype, and promote the production of IL-9 through PPARɣ activation. Interestingly, similar DC2-Th2 crosstalk has also been described in AD regardless of treatment with the IL-4Ra blockade. Therefore, the targeting of pathways that activate and facilitate the persistence of airway mucosal DC2 and Th2 cells could be a new option to induce remission in allergic disease patients [216, 217].

In the periphery, classical DCs (cDCs) are the major type of DC that capture protein antigens that access the epithelial barrier and present them to T cells. cDCs can be further divided into two main subsets: major, or cDC2s, and cross-presenting, or cDC1s. The production of IL-13 and TNF-α by ILC2s and MCs, respectively, may increase the migration of DCs to draining lymph nodes [209]. A recent article reported that in the colon, TSLP can act as a tolerogenic cytokine by regulating the communication between DCs and CD4 + T cells [218], thereby promoting immune tolerance to the gut microbiome. The mechanisms that lead to Th2 polarization are not yet completely understood, but recent findings highlighted that DCs conditioned by epithelial cell-derived alarmins (TSLP, IL-25, and IL-33) generate less production of IL-12 and increase the expression of costimulatory molecules such as OXO40L or Notch receptor ligands because positive feedback skews CD4 + T cells toward Th2 polarization [162, 215, 219]. cDC2s constitute the major DC subset implicated in Th2 differentiation in both mouse models and human in vitro cultures. IRF4 is a key transcription factor required for cDC2 development and survival. Like the IL33 gene, it also regulates pro-Th2 genes. The generation of CD11c-Cre IRF4-floxed mice results in a decreased frequency of Th2 cells during papain immunization, helminth infection, and allergic airway inflammation [164, 220–222].

**Fig. 5:**
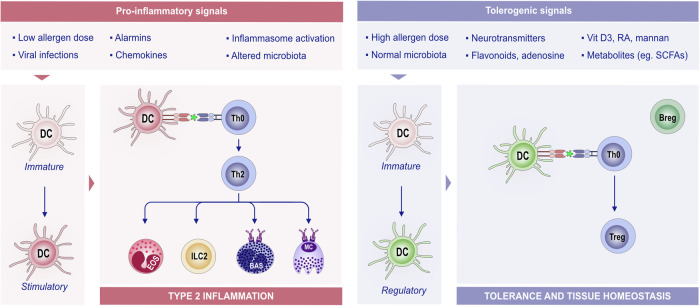
**The role of DCs in type 2 inflammation and tolerance and tissue homeostasis**. DCs are antigen-presenting cells that are able to process and integrate signals from the microenvironment. Upon exposure to proinflammatory stimuli, immature DCs develop into stimulatory DCs and promote an effector immune response by stimulating T-cell proliferation and shaping T-cell responses toward Th2 phenotypes via the indicated signals. DCs play a crucial role in antigen presentation to CD4 + T cells, shaping the subsequent immune response. The interaction between DCs and T cells can lead to different outcomes depending on the antigen dose and environmental signals. For example, a low allergen dose typically primes Th2 cells, promoting the allergic response. A high allergen dose, combined with tolerogenic signals such as vitamin D3 and RA, can induce tolerance. In a tolerogenic environment, DCs acquire regulatory functions that suppress T-cell activation and proliferation and provide signals for Treg differentiation and expansion. Treg and Breg cells support each other’s regulatory functions. These regulatory functions of DCs are key for maintaining immune tolerance and tissue homeostasis. Various factors contribute to this tolerogenic environment, including TSLP, RA, flavonoids, and SCFAs. Bas basophil, Breg regulatory B cells, DC dendritic cells, RA retinoic acid, SCFA short-chain fatty acid, Th2 T helper 2 cells, Treg regulatory T cells, TSLP thymic stromal lymphopoietin

In the presence of a plethora of stimuli, such as neurotransmitters, adenosine, flavonoids, vitamin D3 metabolites, retinoic acid, or mannan, immature or mature DCs can also promote the generation of functional regulatory T (Treg) cells, therefore promoting tolerance and tissue homeostasis (Fig. 5) [223–225].

In the epidermis, Langerhans cells (LCs) share functions with cDCs but are developmentally related to tissue-resident macrophages. LCs may function in the context of skin infections to present external antigens to activate CD4 + T cells or to present CD4 + T cells. LCs are differentiated from other DC subtypes by their expression of CD1a and CD207 and their colocalization with keratinocytes and peripheral sensory neuron terminals [226–228]. LC development from myeloid precursors relies on IL-34, which is produced by neurons and keratinocytes [229, 230]. In humans, blood-derived cDC2s can act as LC progenitors in response to inflammatory signals. The implementation of CD34+ hematopoietic progenitor cells in vitro led to the observation that bone morphogenic protein signaling promotes differentiation into efferocytosis receptor Axl-expressing (Axl + ) cDC2s, which are commonly found to accumulate in psoriatic lesions. Subsequent stimulation with TGF-β1 leads to the production of LCs from Axl+ cDC2s, which mirrors signaling pathways found in human psoriatic epidermal cells [231]. LCs extend their projections throughout the epidermis to sense and guard against infiltrating pathogens to maintain the integrity of the skin barrier [232]. Recently, it has been reported that aryl hydrocarbon receptors activated by dietary ligands reduce allergic skin responses by regulating the migration of LCs [233]. This migration activity may be a potential target for treating allergic skin disease.

**Fig. 6:**
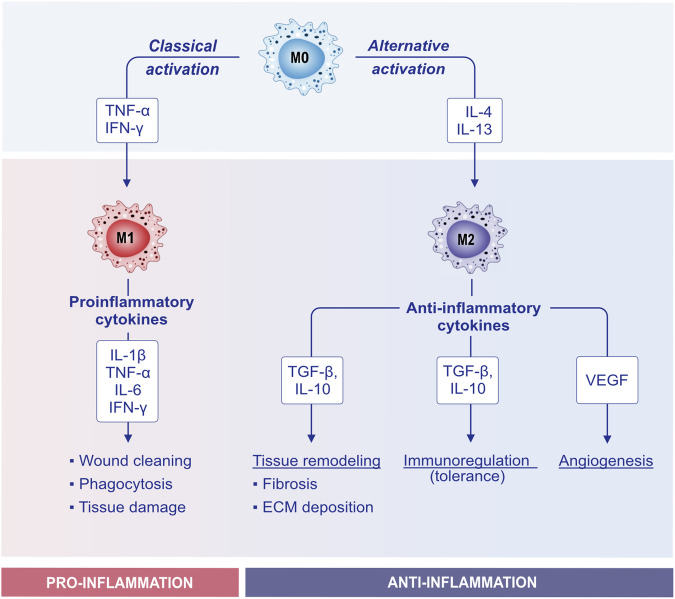
**Activation and polarization of macrophages**. Macrophages adopt different functional states in response to environmental signals. The transition from the M0 (naive) phenotype to the M1 and M2 phenotypes is a key aspect of their plasticity and role in the immune response. Upon proinflammatory triggers such as TNF-α and IFN-γ, M0 macrophages can polarize toward the M1 phenotype, also known as classically activated macrophages, and acquire proinflammatory features. They promote inflammation, and M1 macrophages produce proinflammatory cytokines (e.g., TNF-, IL-1β, IL-6, and IFN-), leading to wound clearance, phagocytosis, and tissue degradation. Alternatively, upon IL-4 and IL-13 signaling, M0 macrophages polarize toward the M2 phenotype or alternatively become activated. M2 macrophages produce IL-10, TGF-β, and VEGF. M2 macrophages contribute to tissue remodeling, immunoregulation (tolerance), and angiogenesis. IFN-γ interferon-γ, IL interleukin, TGF-β transforming growth factor-β, TNF-α tumor necrosis factor-α, VEGF vascular endothelial growth factor

Macrophages represent another class of APCs. The macrophages that reside in the lung contribute to maintaining the homeostasis of the organ by patrolling airways and removing dead cells, inhaled particles, and external invaders (e.g., bacteria). Macrophages are key orchestrators of the immune response by recruiting eosinophils, neutrophils, and monocytes. Persistent airway thickening and remodeling consists of increased airway epithelial thickening, mucus hypersecretion, airway smooth muscle mass, and collagen deposition with the consequent restriction of airflow [234, 235]. Macrophages secrete factors that promote airway remodeling, such as IL-4 and IL-13, and the profibrotic growth factors TGF-β and PDGF [236]. Macrophages can be roughly divided into two different subsets: M1 (proinflammatory) and M2 (anti-inflammatory). M0 macrophages can undergo classical activation through stimulation with TNF-α and IFN-ɣ, which leads them to polarize toward the proinflammatory M1 phenotype. Conversely, upon stimulation with IL-13 and IL-4, M0 macrophages undergo alternative activation that polarizes them toward the M2 phenotype (Fig. 6) [237]. In recent years, the importance of M2a macrophage activation in allergic asthma has been indicated [238–240]. IL-4 and IL-13 interact with the IL-4 receptor and activate signal transducer and activator of transcription 6 (STAT6), inducing M2a macrophage activation [241]. A recent study reported that circular RNA (circS100A11) and S100A11 promote M2a macrophage activation and lung inflammation in an asthma model. These findings may serve as potential therapeutic and diagnostic targets for children with asthma [242].

Interestingly, a recent study investigated the interaction between the epidermis and macrophages during embryonic skin development. The authors state that the epidermal stress in Integrin B1 knockout mice triggers the recruitment of monocyte-derived macrophages to the skin, rather than favoring the proliferation of resident macrophages. These recruited macrophages are characterized by the expression of both M1 (proinflammatory) and M2 (pro-remodeling) markers. Therefore, this study revealed complex crosstalk between the stressed epidermis and macrophages, leading to sterile inflammation and excessive extracellular matrix deposition in the embryonic skin [243]. In the context of skin repair, resistin-like α (RELMα) secreted from IL-4- and IL-13-activated macrophages activates fibroblasts, which leads to the upregulation of lysyl hydroxylase 2, an important protein for the regulation of the mechanical crosslinking of collagen fibrils [244].

Other studies have suggested that IL-4 and IL-13 may not be sufficient to activate tissue repair macrophages. Indeed, other reports have shown that IL-33 signaling may be important for remodeling by IL-4- and IL-13-activated macrophages that occur in adipose tissue and the lung. Taken together, these examples illustrate the wide range of roles of macrophages in the different organs that regulate inflammation, repair, and fibrosis [235, 245–247].

### Type 2 innate lymphoid cells

Tissue-resident cells such as epithelial cells, ILCs, mast cells, and dendritic cells form a complex network of multicellular cooperation and play crucial roles in generating the type 2 immune response. ILCs are innate counterparts of lymphoid cells and lack rearranged antigen receptors and markers for myeloid and conventional lymphoid lineages. Depending on the type of immune response they participate in, there are three distinct helper-like ILC subpopulations: ILC1s, ILC2s, and ILC3s. Like those produced by Th1, Th2, and Th17 cells, the main cytokines produced by these ILCs are the type 1 cytokine IFN-γ; the type 2 cytokines IL-5 and IL-13; and the type 3 cytokines IL-17 and IL-22. In addition, there are other types of ILCs, such as NK ILCregs, which are lymphoid tissue-inducer cells [248–250]. ILCs are generated from primitive hematopoiesis originating in the yolk sac and liver and from bone marrow hematopoietic stem cells [251]. They reside in the small intestine lamina propria, lung, dermal, and submucosal fascial planes in the skin, liver, salivary gland, bone marrow, secondary lymphoid organs, and peripheral blood [252]. For ILC2 development, IL-7, IL-33, and Notch signaling are needed. Their differentiation and maintenance are regulated by GATA-3. The transcription factor RORα regulates the differentiation of ILC2s from their progenitors [158, 253].

The ILC2 subset is the main source of early IL-5 and IL-13 secretion in tissue activated by the alarmins IL-25 and IL-33 to elicit type 2 immunity [251]. Importantly, the frequency of ILCs is decreased during allergen immunotherapy [254]. Another alarmin, TSLP, is important for ILC2 survival [255] and activation, especially in the skin [158, 251]. Prostaglandin D2 regulates ILC2 migration and type 2 cytokine production through its receptor, chemoattractant receptor-homologous molecule expressed on Th2 cells (CRTH2). CRTH2 activation induces increased IL-25 and IL-33 receptor subunit expression [256].

ILC2s are essential for maintaining epithelial barrier homeostasis because they aid in the repair and regeneration of tissue by regulating tissue-resident stem cells while sustaining adequate inflammation in pathogens such as parasitic helminths. The capacity of ILC2s to maintain tissue homeostasis in response to external stimuli is well documented in the small intestine. Luminal succinate, which is secreted by *Tritrichomonas muris*, a commensal in the mouse intestine, activates tuft cells to secrete IL-25, which induces ILC2 activation in the lamina propria. In turn, activated ILC2s secrete type 2 cytokines and amphiregulin, a well-known growth factor that induces both mitogenesis and cell differentiation [257–260]. Their lack causes significant deficiencies in the type 2 immune response, as shown by mouse models [261].

Despite their important roles in maintaining tissue homeostasis and controlling parasitic infections, they play a role in allergic diseases. IL-33-activated ILC2s are known to contribute to virus-induced bronchial hyperreactivity and allergic asthma. Following allergen challenge, ILC2s accumulate in the sputum of patients with severe eosinophilic asthma. ILC2s contribute to the characteristic features of asthma, such as mucus secretion, smooth muscle contraction, and infiltration of inflammatory cells through the secretion of type 2 cytokines. ILC2s are enriched in nasal polyps from chronic rhinosinusitis patients and skin biopsies from atopic dermatitis patients and are among the main producers of IL-5 and IL-13 in the lungs of asthmatic patients [158].

Environmentally toxic agents such as air pollutant particulate matter and detergents cause ILC2 activation through alarmin release [262]. The activation of ILC2s in the midline and mucosal barrier sites causes epithelial barrier impairment via type 2 cytokines such as IL-13. It has been shown that laundry detergent exposure increases IL-33 expression through ROS induction, which results in subsequent ILC2 activation and type 2 inflammation [28]. A recent study revealed that TSLP release due to *S. aureus*-released indole-3-aldehyde primes ILC2s and increases susceptibility to allergic diseases [263].

Metabolic perturbations critically affect ILC2 fate and effector functions, such as differentiation, migration, plasticity, and activation [225, 264, 265]. Most evidence in the field comes from mouse models, showing that ILC2s rely on fatty acid oxidation to fuel oxidative phosphorylation (OXPHOS) and meet their energetic demands [264, 265]. Forcing a switch from OXPHOS to aerobic glycolysis reduces the secretion of IL-5 and IL-13 in ILC2s [264]. Fatty acids (FAs) are essential for the generation, expansion, and activation of ILC2s. Peroxisome proliferator-activated receptor gamma (PPAR-γ) regulates FA uptake, as demonstrated by decreased expression of the FA transporter CD36 upon its pharmacological inhibition in tissue-resident ILC2s in the lung and adipose tissue [266, 267]. In the context of allergen-induced lung inflammation, in the inflammatory microenvironment, ILC2s enhance the absorption of both FAs and glucose. FAs obtained externally are stored in lipid droplets and transformed into phospholipids, aiding the proliferation of ILC2s. Importantly, eliminating PPAR-γ in ILC2s diminished the uptake of external FAs and the formation of lipid droplets by reducing the expression of triglyceride-synthesizing enzyme (DGAT1). This resulted in impaired proliferation of ILC2s and decreased production of IL-5 and IL-13 [267]. In addition to FA metabolism, amino acid (AA) metabolism is crucial for the development and functionality of ILC2s. The absence of Arginase 1 (Arg1) disrupts the AA balance, subsequently reducing immune responses, proliferation, and cytokine production [264, 265]. During helminth infection, ILC2 functions depend on the large neutral AA transporters *Slc7a5* (LAT1) and *Slc7a8* (LAT2), whose deletion impairs effector functions partially via the mTOR pathway [268]. Notably, a recent study revealed that programmed death protein-1 (PD-1) acts as a metabolic checkpoint for ILC2s, inhibiting their effector functions, such as cytokine production and survival. PD-1 deficiency shifts ILC2s toward glycolysis, as well as methionine and glutamine catabolism [269].

Much less is known about the immunometabolism of ILC2s in humans. Surace et al. [270]. demonstrated that human ILC2s utilize dichotomous metabolic pathways to support their fate and functions. Circulating “naive” ILC2s depend on electron transport chain complexes I and III for their survival and rely on branched-chain amino acids and arginine to fuel OXPHOS. Upon activation with IL-33, ILC2s become highly glycolytic, depending on the mTOR pathway, to produce IL-13 and simultaneously fuel OXPHOS with AAs to maintain cellular fitness [270].

Importantly, the metabolic regulation of ILC2s can be influenced by various factors, including the organism being studied (mice vs. humans), the type of trigger (helminth vs allergen), the localization of ILC2s (circulating vs. tissue-resident), and their specific microenvironment (lung, gut, or skin). Further research is necessary to fully understand the metabolic regulation of ILC2s in humans.

### Mast cells in the type 2 response

As innate granulocytes, MCs—together with eosinophils and basophils—act as specific effectors in type 2 immune responses, are capable of activating and modulating ILC2 functions and quickly release substantial amounts of preformed mediators such as cytokines, proteases, lipid mediators, and histamine under the influence of type 2 cytokines [271, 272]. Prostaglandins and leukotrienes, significant modulators of ILC2 function, are expressed at high levels because of myeloid cell recruitment and mast cell activation [273].

MCs are enduring constituents of the immune system that engage promptly at the onset of infections, facilitate helminth clearance in later stages, and substantially contribute to protective immune responses during secondary infections [274]. IL-33 stimulates MC activation and proliferation, leading to degranulation and the release of preformed mediators that modulate cells of both the innate and adaptive immune systems. These mediators include IL-4 and IL-13, which drive alternative activation of macrophages [275]; prostaglandin D2, which cleaves IL-33 and enhances ILC2 induction through CRTH2 receptor interaction [276]; and TNF-α, CXCL1, and CXCL2, which promote the recruitment and proliferation of neutrophils at the infection site [277, 278]. This series of events highlights the key role of IL-33 in driving immune responses through MC pathways. MC degranulation provokes effects such as goblet cell hyperplasia and increased mucin production, which together enhance peristalsis and create a helminth-hostile environment. Additionally, MCs serve immune regulatory functions and act as key effectors of the inflammatory response through degranulation [279]. In the context of barrier damage, MCs, along with IgE, play a role in the development of allergic diseases as part of Type 2 immune responses [280]. Additionally, MCs can regulate immune niches by recruiting macrophages and modulating their polarization, thereby impacting disease progression [281].

## Regulation and Functions of IgE and IgG4 Antibodies in the Type 2 Response

IgE plays a crucial role in allergic sensitization and contributes to inflammation driven by MCs and basophils. IgE production from B cells is regulated by IL-4 and IL-13 [282]. Low-affinity IgE production occurs from direct class switching from μ to ε with less mutation. In contrast, high-affinity IgE production is the result of sequential class switching from μ to γ to ε characterized by an intermediate IgG phase and somatic hypermutation [283]. IgE memory is primarily found in rare IgE memory B cells and mainly in IgG1+ memory B cells that convert to IgE upon re-exposure to antigens [284]. Antigen-specific IgE antibodies to allergens, such as house dust mites, animal dander, molds, milk, eggs, fish, peanuts, and drugs, can bind to MCs and basophils through the FcεRI [285, 286]. When these allergens are recognized by specific IgE antibodies on the surface of the MC membrane via the spleen tyrosine kinase (Syk), the release and production of various substances are triggered. Most of these immediate mediators of a type 1 reaction, including lipid mediators, such as leukotrienes and prostaglandin PGD2, histamine, proteoglycans, proteases, and proteoglycans, have been previously synthesized and stored inside MCs [287]. With the activation of MCs, the synthesis of cytokines, such as IL-4, IL-5, IL-13, TSLP, TNF-α, and TGF-β1, is induced [287, 288]. This induces vasodilation and increased vascular endothelial permeability, leading to hypovolemic shock and anaphylaxis [287]. There are several strategies to inhibit this cascade, including the targeting of IgE antibodies with anti-IgE monoclonal antibodies, the design of ankyrin repeat proteins (DARPins) to inhibit FcεRI–IgE interactions, the binding of fusion proteins to FcεRI with the inhibitory FcγRIIb, Syk inhibition of glucocorticosteroids and antihistaminic treatment [289–292]. Anti-IgE mAb-based treatment has been shown to be beneficial for allergic diseases, asthma, chronic urticaria, food allergies, allergic rhinitis, and allergic bronchopulmonary aspergillosis and can decrease the adverse effects of allergen immunotherapy [292–297].

In addition to the very well-defined classical role of IgE in the allergic inflammatory cascade, compelling experimental evidence indicates that IgE also significantly contributes to the pathogenesis of other diseases, such as autoimmune diseases and cancer [298, 299]. The role of IgE in nonclassical allergic diseases such as chronic spontaneous urticaria (CSU) [300] and CRSwNP [301] is supported by the clinical efficacy and safety of omalizumab, an anti-IgE monoclonal antibody approved for these diseases. Patients suffering from CSU display positive serum levels of autoreactive IgE antibodies against autoallergens and/or IgG-anti-IgE, IgG-anti-FcεRI or both, leading to type I or type II autoimmunity, respectively [2, 300]. CSU patients with autoreactive IgE respond faster to omalizumab treatment than those with IgG-anti-IgE or anti-FcεRI antibodies do, contributing to the better stratification of CSU patients [302]. IgE also plays a relevant role in different autoimmune diseases, including organ-specific diseases such as bullous pemphigoid, Grave’s disease, Hashimoto disease, and autoimmune uveitis, as well as in systemic diseases such as systemic lupus erythematosus (SLE), mixed connective tissue disease, Gougerot-Sjögren syndrome, and systemic sclerosis [298]. Although the molecular mechanisms underlying the actual role of IgE in autoimmunity are not yet fully understood, our knowledge has significantly improved in recent years. In atopic dermatitis, IgE antibodies might be generated either against autoantigens or against exogenous allergens [298, 303]. IgE against exogenous allergens might cross-react with self-antigens due to molecular mimicry, also contributing to autoimmunity, worsening and disease progression [298, 303]. Autoreactive IgE antibodies induce the activation and degranulation of different effector cells, such as mast cells, basophils or eosinophils, upon encountering autoantigens, thus leading to the release of proinflammatory mediators that contribute to tissue damage and to the recruitment of inflammatory cells to target organs [304]. In SLE, specific IgE antibodies bind to dsDNA, generating immunocomplexes that are recognized and internalized into endosomal compartments by plasmacytoid dendritic cells (pDCs) through the high-affinity IgE FcεRI, which leads to TLR9-mediated potent interferon and proinflammatory responses, contributing to self-destructive autoimmunity [305]. In contrast, IgE-mediated cross-linking of FcεRI on pDCs impairs TLR9-mediated IFN-alpha production, whereas TLR9 activation downregulates the expression of FcεRI [306, 307]. These data suggest that IgE might play a dual role in SLE depending on whether IgE‒dsDNA immunocomplexes are internalized or whether nonautoreactive IgE‒mediated cross-linking of FcεRI takes place in pDCs from patients with SLE. Interestingly, IgE cross-linking on pDCs from atopic donors impairs their capacity to generate Tregs in vitro, which can be restored by anti-IgE monoclonal antibodies such as omalizumab or ligelizumab [306, 308]. Further research is needed to assess whether this mechanism also contributes to inflammatory responses in other autoimmune diseases in which high IgE levels are reported, such as Crohn’s disease and other inflammatory bowel diseases. The capacity of IgE to increase antigen uptake by APCs might also contribute to breaking tolerance in autoimmunity, which in turn favors the generation of pathological autoantigen-specific T cells rather than suppressive Tregs [304]. IgE can also regulate B cells and promote sustained IgE levels and epitope spreading via mechanisms involving CD23, the IgE low-affinity receptor [2, 298].

Compelling experimental evidence indicates that IgE might also play key functional roles in the context of cancer. Mechanistic studies and substantial epidemiological data support that IgE, allergy, and atopy might confer antitumor and immunosurveillance functions, thus protecting against specific tumor types [299, 309]. In contrast, other studies reported the opposite data, suggesting that IgE and chronic type 2 inflammation might confer protumoral effects [299, 309]. Although the actual nature of the relationship between IgE-associated immune responses and cancer and their potential consequences for cancer development and prognosis remain controversial, four different hypotheses underlying antitumor or protumor effects have been proposed [309]. Two hypotheses support the potential antitumor effects of IgE: i) the immune surveillance hypothesis, which considers atopy as a condition associated with chronic enhanced immune responses able to detect and eliminate potential altered precancerous cells [310]; and ii) the prophylaxis hypothesis, which suggests that the typical clinical signs and symptoms of allergic diseases (i.e., coughing, sneezing, mucus production or itching) help to expel potential carcinogens and to enhance tissue repair [311, 312]. On the other hand, two hypotheses support the potential protumor effects of IgE: iii) the chronic inflammation hypothesis, which suggests that IgE-mediated allergic inflammation is associated with oxidative stress, gene mutations and tissue remodeling, thus increasing cancer risk [310]; and iv) the Th2 skewing hypothesis, which suggests that enhanced Th2 responses impair antitumor Th1 immune responses, thus promoting local tissue environments that are permissive for cancer development [299]. Local permissive tumor environments might favor the growth of cancer cells expressing aberrant carbohydrates that are able to promote the generation of suppressive Treg cells, contributing to tumor growth and metastasis [313, 314]. Considering the complexity of the contribution of IgE to cancer and the multifactorial components influencing an individual’s risk of developing a specific cancer, a combinatorial hypothesis has also been proposed [299, 309]. This hypothesis aims to integrate all four previous methods by considering the different local and systemic immune responses generated in allergic diseases that might influence the risk for the development of different cancer types in individual patients [299, 309]. Finally, supported by epidemiological findings showing that high levels of IgE might confer protection against certain cancer types and considering the broad effector responses triggered by IgE on different cell types (including mast cells, basophils, neutrophils, eosinophils, macrophages or monocytes), the use of IgE-based therapeutic strategies targeting specific tumor antigens has been proposed as a potential novel strategy to complement the already established IgG class antibodies [299, 309]. In this context, a phase I dose escalation trial (NCT02546921) demonstrated the safety and tolerability of MOv18 IgE, a chimeric first-in-class IgE antibody, in patients with tumors expressing folate receptor-alpha, thus supporting the potential of IgE therapy for cancer [315].

The effects of type 2 immunity on cancer are complex and are heavily dependent on the tumor stage, type and corresponding microenvironment [316–318]. Effects correlating with diminished cancer growth are often observed in conjunction with eosinophilic infiltration into the tumor tissue [319, 320]. They seem to play an active role in the clearance of tumor cells and are dependent on their effectiveness in type 2 cytokine signaling induced by the tumor microenvironment [321, 322]. In particular, GM-CSF, IL-4 and IL-5, which are produced by tumor-infiltrating ILC2s [323, 324], are crucial for the recruitment and cytotoxicity of eosinophils in tumor models [325, 326]. Neutralization of IL-4 and IL-5 results in increased tumor load and impaired clearance [318]. IL-33 activates eosinophils to produce and secrete granzymes to eliminate tumor cells [321, 327]. Additionally, IFN-γ supports eosinophilic cytotoxicity [321], and stimulated eosinophils acquire M1-like transcriptional profiles that have antitumor functionality [328]. However, there are also multiple reports that type 2 immunity promotes tumor growth or persistence. Th2 cells and eosinophils produce IL-8 and VEGF in response to TSLP and promote angiogenesis, aiding tumor growth [329]. TSLP can promote tumor growth by acting as an antiapoptotic agent, inducing B regulatory cells, inhibiting type 1 antitumor immunity and promoting metastasis [330]. It also promotes the production of IL13 and TNF, aiding in type 2 immunity induction and type 1 immunity suppression [331]. Conversely, there have also been reports that TSLP prevents skin carcinoma and has positive effects on colorectal and breast cancer [332, 333].

In contrast to specific IgE functions, antigen-specific IgG4 has particular features that make it an anti-inflammatory antibody in the context of the allergen response and type 2 inflammation. Among these features is its dynamic nature due to fragment antigen-binding (Fab)-arm exchange, which results in bispecificity and monovalence against allergens [334–336]. Through this mechanism, IgG4 can compete for binding sites with IgE and inhibit cross-linking of the allergen on effector cells such as basophils and/or mast cells, which express surface FcεRI and FcgRIIb [337]. Other additional properties include low binding affinity to C1q and Fcg receptors, which make them incapable of fixing immune complexes and activating the complement pathway [335, 338].

Allergen-specific immunotherapy (AIT) is currently the only treatment option available that can change the course of IgE-mediated disease through exposure to a given dose of allergen in a controlled manner [339]. Although the full mechanisms of tolerance induced by AIT have not yet been elucidated, the increase in regulatory populations, such as regulatory T and B cells, and the production of cytokines with immunoregulatory properties reduce type 2 (T2) responses and shift toward the production of antigen-specific protective antibodies, especially IgG4 [340, 341].

In vivo models of natural tolerance to high-dose allergen exposure (e.g., beekeepers and helminth infections) have demonstrated an increase in IgG4 titers [342, 343] induced by the presence of interleukin (IL)-10-secreting type 1 T regulatory (Tr1) cells [344, 345]. Both IgE and IgG4 share similarities in epitope recognition [346–348] but also in the conditions that induce their production [349]. Importantly, however, the presence of IL-10 is fundamental for the induction of IgG4 production [350, 351]. Additionally, there is possible “loop feedback”, where regulatory B cells (Br1) with increased expression of IgG4 and IL-10 have been described as being able to suppress the inflammatory response through IL-10 production and expand after allergen exposure [352, 353]. Furthermore, class-switched IgG4 B cells are not a single subset. A particular population has been described in eosinophilic esophagitis and melanoma, which have distinctive markers, such as CD49b and CD73, and proangiogenic properties through cytokine production [354].

Although IgG4 is the most recognized antibody with IgE blocking capacity, other isotypes, such as IgD and IgG2, have also demonstrated similar capacities explained by alternative mechanisms; however, there is less evidence [341, 355, 356].

## Alarmins and Type 2 Immunity

### Epithelial stimulation and barrier defects initiate a type 2 immune response

**Fig. 7:**
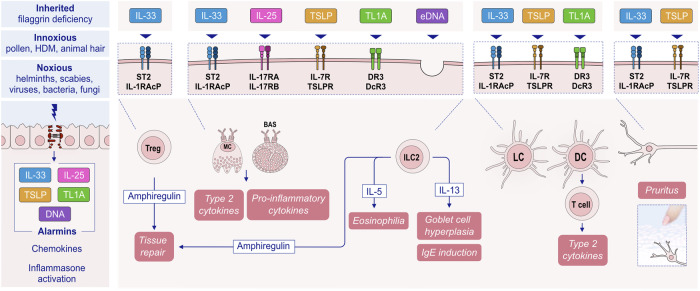
**Immune and inflammatory responses to allergens**. Exposure to various triggers, such as protease allergens, helminths, fungi, and viruses, leads to the release of the alarmins IL-25, IL-33, TSLP, TL1A and eDNA by epithelial cells. TSLP, IL-25, IL-33, TL1A, and eDNA activate ILC2s, promoting the production of the type 2 cytokines IL-5 and IL-13. ILC2s also promote eosinophilia, goblet cell hyperplasia, and IgE production via immunoglobulin class switch recombination in B cells. DCs and LCs are activated by TSLP to stimulate allergen-specific Th2 cells. Mast cell and basophil degranulation, along with cytokine production, is increased by IL-33 and IL-25 following IgE cross-linking. Furthermore, ILC2s facilitate fibrosis and tissue repair through IL-5, IL-13, or amphiregulin. TSLP and IL-33 also directly stimulate itch-sensory neurons, leading to pruritus. Bas basophil, DC dendritic cell, eDNA extracellular DNA, DR3 death receptor 3, DcR3 decoy receptor 3, IL interleukin, ILC2 type 2 innate lymphoid cell, IL-1RAcP IL-1 receptor accessory protein, LC Langerhans cell, MC mast cell, Th2 T helper 2 cell, TSLP thymic stromal lymphopoietin

**Fig. 8:**
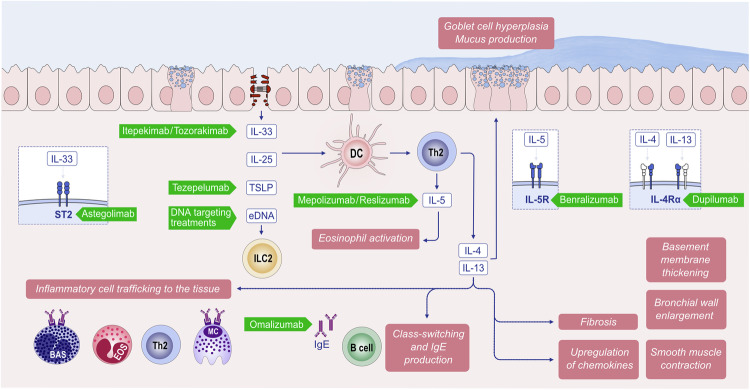
**Existing and emerging biological treatments for asthma**. Mepolizumab and reslizumab target IL-5. Benralizumab targets the α chain of the IL-5 receptor. All three antibodies lead to the suppression of eosinophil activation and number. Omalizumab functions as an antibody that inhibits IgE. Dupilumab interacts with the α subunit of the interleukin-4 receptor, inhibiting signaling pathways for the type 2 cytokines IL-4 and IL-13. Itepekimab and tozorakimab target IL-33, and astegolimab targets ST2, the IL-33 receptor. Tezepelumab targets TSLP. There are several new treatments in development that target DNA. IL, interleukin; TSLP, thymic stromal lymphopoietin

Type 2 immunity is induced not only by noxious stimuli but also by common household chemicals, pollen, mites, and airborne particulate matter, which are known to worsen asthma [17, 357]. This finding is especially relevant, as T2 cytokines increase susceptibility to viral infections in patients with asthma, not only by changing the structure of the epithelium but also by modulating the expression of viral entry receptors and the production of IFNs. Notably, allergen immunotherapy (AIT) can reduce viral exacerbation rates in patients with asthma by increasing the expression of type I/III IFNs and decreasing the expression of IL-33 [49, 358–361]. Additionally, inherited defects in genes necessary for protective barrier function can exacerbate this condition by compromising the integrity of these barriers, leading to heightened sensitivity and an increased likelihood of developing allergies and other related disorders [362, 363]. Different immune cells coordinate responses at these sites by interacting with each other and with nonimmune cells, such as epithelial cells [364, 365]. Type 2 immune responses occur in response to parasitic helminths (e.g., nematodes) and insects (e.g., scabies), restricting sites permissive for parasite and insect reproduction and limiting barrier tissue damage. These responses involve the activation of ILC2s and Th2 cells, which secrete the IL-4, IL-5, and IL-13 necessary to accumulate eosinophils and, alternatively, activated macrophages in involved tissues [362, 366]. Epithelial barrier disruption and epithelial cell damage trigger inflammation by releasing alarmins such as IL-25, IL-33, TL1A, and TSLP and damage associated molecular patterns such as extracellular DNA (eDNA), leading to the development and exacerbation of allergic and type 2 diseases [367] (Fig. 7). Alarmins can directly activate ILC2s and Th2 cells to produce type 2 cytokines, but they do not have the same effect on pathogenesis across all type 2 diseases [55, 368]. TSLP and IL-33 signaling mutually enhance each other’s protein release and expression in the lung after allergen exposure, and they also increase each other’s receptor expression on lung ILC2s, increasing ILC2 activation [25]. Particulate matter exposure increases the release of the alarmin cytokines TSLP, IL-33 and IL-25 from epithelial cells by causing direct DNA damage, tight junction disruption and increased oxidative stress [369]. Viral infections, another well-known trigger of asthma, have also been shown to increase the release of IL-25, IL-33 and IL-1β from epithelial cells [49, 370, 371]. Moreover, IL-33 and TSLP directly activate MCs. TSLP stimulates Th0 cell differentiation toward Th2 cells through stimulation by DCs. TSLP also promotes B-cell proliferation [53, 372]. TL1A has been demonstrated to play a crucial role in promoting inflammation by inducing Th2/IL-13 mucosal responses and activating T cells to produce IL-5 and IL-13. eDNA combined with Alternaria allergens enhances the immune response by increasing IL-5 and IL-13 levels in the lung. These alarmins that effectively identify disease endotypes or phenotypes may also serve as therapeutic targets [373] (Fig. 8).

### IL-25 and type 2 immunity

IL-25 binds to a heterodimeric receptor consisting of IL-17RA and IL-17RB and activates the MAPK (JNK and p38) and NK-κB pathways. In contrast to T and B cells, ILC2s are directly stimulated by epithelial cytokines such as IL-25. IL-25-deficient mice struggle to effectively expel *Nippostrongylus brasiliensis* because of altered Th2 cytokine release and are highly susceptible to experimental autoimmune encephalomyelitis [53]. Mice with transgenic expression of IL-25 exhibit eosinophilia and elevated levels of IgE, IgG1, IL-5, and IL-13. IL-25 is constitutively expressed in tuft cells (also called brush cells) in the intestine and trachea during inflammation [374, 375].

IL-25 may also be involved in the pathogenesis of asthma. In mice, intranasal administration of IL-25 alone induces ILC2-mediated type 2 inflammation in the lung. IL-25 is one of the major cytokines responsible for airway remodeling in asthma. Subepithelial collagen and fibronectin deposition is increased by IL-25 [376]. Neutralizing IL-25 in the lungs with a soluble IL-25R fusion protein has been shown to inhibit antigen-induced CD4 + T-cell recruitment into the airways, IL-5 and IL-13 production, and goblet cell hyperplasia [377].

### IL-33 and type 2 immunity

IL-33, a member of the IL-1 family of cytokines, is constitutively expressed at high levels in the nuclei of epithelial cells. After epithelial cell injury or cellular activation through ATP signaling, these cells promptly release IL-33 [378]. After release, IL-33 needs to be proteolytically cleaved to become bioactive. The protein can be cleaved by allergen proteases or proteases released by inflammatory cells [379]. The ripoptosome, an intracellular molecular signaling platform that can also drive the maturation and secretion of IL-33, is activated in response to epithelial barrier disruption by allergens, initiating type 2 innate immune responses [380]. Upon release, mature IL-33 binds to a heterodimeric receptor formed by IL-1 receptor-like 1 (IL1RL1), also known as ST2, and the IL-1 receptor accessory protein, which leads to the activation of structural and immune cells through the NF-κB and MAPK (ERK, p38, JNK) signaling pathways [378, 381]. The genes encoding IL-33, ST-2, and IL1R1 are among the few genes that have been shown to be associated with asthma [382, 383]. Although already present at high levels in the steady state, the expression of IL-33 can be further increased during inflammation, such as in COPD, graft-versus-host disease, helminth infection, and AD [378].

The first evidence for the role of IL-33 in host defense was in helminth infection [384]. IL-33, together with IL-25 and TSLP, mediates worm expulsion via the activation of MCs and ILC2s, which increases IgA production via IL-6 and enhances goblet cell hyperplasia via IL-13. Consistently, human serum IL-33 levels are correlated with clinical asthma and AD severity. The administration of IL-33 to mice led to airway inflammation through the activation of ILC2-producing type 2 cytokines that mobilize eosinophils and polarize alternatively activated macrophages. ST2-deficient mice exhibit normal maturation of Th2 cells but altered antigen-specific Th2-type responses, as well as increased rates of ventricular fibrosis and cardiomyocyte hypertrophy in response to ventricular pressure overload. Compared with wild-type mice, IL-33-deficient mice presented attenuated eosinophilic pulmonary inflammation in an OVA-induced asthma model. However, the serum OVA-specific IgE levels were comparable between these two groups [385–388]. IL-33-deficient mice also exhibited attenuated eosinophilic pulmonary inflammation in a protease-induced asthma model, which was independent of T and B cells. IL33-IL1RL1 pathway polymorphisms are associated with asthma and specific wheezing phenotypes; most single nucleotide polymorphisms are associated with intermediate-onset wheezing, a phenotype closely related to allergic sensitization [389]. Compared with placebo, tepekimab is a new monoclonal antibody that has been reported to decrease the incidence of asthma control loss events and improve lung function in patients with moderate-to-severe asthma [390]. In preclinical studies, tozorakimab demonstrated potent inhibition of IL-33-driven inflammatory responses and showed potential for reducing inflammation and promoting epithelial repair [391]. In addition, astegolimab, an inhibitor of the ST2 receptor, has been shown to significantly reduce the rate of asthma exacerbations in adults with severe asthma, including individuals with low eosinophil counts [392]. On the other hand, IL-33 can induce the resolution of inflammation and the repair of tissue damage. This is likely due to the ability of IL-33 on Tregs and ILC2s to produce amphiregulin, which in turn supports epithelial tissue repair [393]. Thus, IL-33 plays an essential role in the induction of type 2 immunity and the resolution of inflammation.

### TSLP and type 2 immunity

TSLP is a member of the IL-2 cytokine family and was initially identified as a molecule that can stimulate murine thymocytes and promote B-cell proliferation and development [394]. TSLP is produced by epithelial cells at barrier surfaces upon mechanical injury (such as scratching), exposure to protein allergens with protease activity (such as pollens and mites), and exposure to cytokines (e.g., TNF-α and IL- 1β) [395]. TSLP is found as two isoforms in humans, derived from the same gene as a result of expression from different promoters. The short isoform of TSLP, which is predominantly expressed in epithelial cells, is responsible for maintaining immune homeostasis in the gut and lungs [396, 397], whereas the long isoform is related to Th2-mediated immunity/inflammation in the lungs and skin [397, 398]. In AD and asthma, TSLP is highly expressed in the epithelium of the skin and lungs, respectively. TSLP binds to a heterodimeric receptor consisting of the IL-7 receptor (IL-7R) α-chain and the TSLPR chain. In mice, the TSLP-signal transducer and activator of transcription (STAT) 5 axis in DCs is a critical pathway that promotes type 2 immune responses at barrier surfaces [399].

Cigarette smoke, a well-known aggravator of asthma, has been demonstrated to increase TSLP secretion from bronchial epithelial cells [400]. Under inflammatory conditions, TSLP induces DCs to upregulate costimulatory molecules such as OX40L, CD80, and CD86 [401], which then drive IL-4, IL-5, and IL-13 production by CD4^+^ T cells in vitro. In vivo, TSLP-TSLPR signaling in epidermal Langerhans cells is critical for inducing Th2-type immune responses in an OVA application-induced AD mouse model [402]. TSLP can also directly act on circulating CD4^+^ T cells in AD patients, which show increased expression of the TSLPR compared with healthy subjects; the frequency of circulating TSLPR + CD4 + T cells correlates with serum CCL17/TARC and IgE levels and eosinophil counts [403]. TSLP also increases the survival of eosinophils in tissue by upregulating the expression of adhesion molecules to fibronectin [404]. Additional pathways by which TSLP promotes type 2 immunity involve acting on MCs, ILC2s, epithelial cells, macrophages, and basophils [405]. Tezepelumab, a human monoclonal antibody that targets TSLP to block its interaction with the heterodimeric receptor, is the leading antialarmin in advanced clinical development aimed at treating asthma [406].

Keratinocyte-derived TSLP directly activates transient receptor potential cation channel subfamily A member 1 channels on sensory neurons [407], which exacerbates pruritus. Similarly, IL-31 released by type 2 immune cells in skin lesions mediates pruritus by stimulating neurons expressing IL-31 receptor subunit α (IL-31Rα) [408], increasing neurite branching in the skin [409]. In a phase 2 trial, a humanized monoclonal anti-IL-31Rα antibody significantly improved pruritus in AD patients [410].

### TL1A and type 2 immunity

TL1A, a member of the TNF superfamily secreted from epithelial cells, has been shown to play a significant role in triggering Th2 responses at the onset of allergic air inflammation [411]. It plays a crucial role in promoting inflammation by inducing Th2/IL-13 mucosal responses, which are significant contributors to conditions such as colitis [412]. In the context of inflammatory autoimmune diseases, TL1A has been demonstrated to activate T cells, producing IL-5 and IL-13, which are characteristic cytokines of Th2 cells [413]. Furthermore, TL1A has been shown to directly stimulate ILC2s to produce type 2 cytokines independently of other stimuli, such as IL-25 or IL-33, further emphasizing its role in promoting Th2 responses [414].

TL1A has been implicated in inducing the expression of IL-13 by innate lymphoid cells, leading to mucus production, airway inflammation, and fibrosis, all of which are characteristic features of asthma [415]. In conditions such as asthma, TL1A contributes to a Th2-dominant environment that favors fibrosis and tissue remodeling, highlighting its role in disease progression from moderate to severe stages [416]. Moreover, TL1A has been linked to the generation of pathogenic Th9 cells in inflammatory bowel disease, further highlighting its involvement in driving specific Th2 cell responses in various pathological conditions [417].

### Other DAMPs and type 2 immunity

Severe epithelial damage and even stimulation of the epithelium by allergens can lead to the release of eDNA [418, 419]. This release of eDNA, which can be either nuclear (nDNA) or mitochondrial DNA (mtDNA), can amplify inflammatory responses in the airways. In patients with treatment-resistant severe asthma, airway inflammation is associated with eDNA within bronchial biopsy samples or sputum samples, which is correlated with decreased lung function and asthma control [419, 420]. In mouse asthma models, *Alternaria* can induce the release of both nDNA and mtDNA from epithelial cells, whereas the house dust mite (HDM) antigen triggers the release of only mtDNA. eDNA with *Alternaria* allergens amplified the immune response through increased IL-5 and IL-13 levels in the lung [418]. The only FDA-approved drug that targets extracellular DNA is Dornase alfa, which was originally developed to treat cystic fibrosis and could be a promising treatment for the early development of asthma.

The C-type lectin receptor Mincle (macrophage-inducible C-type lectin) is primarily recognized for its ability to identify non-self-glycolipids in the cell walls of bacteria and fungi, as well as to detect self-damage by recognizing SAP-130 [421]. Following epithelial barrier damage, cholesterol sulfate, which is abundant in epithelial cells, is released. This release of cholesterol sulfate enhances the type 2 immune response in the skin through its interaction with the Mincle receptor [422]. However, dietary supplementation with cholesterol sulfate alleviated dextran sodium sulfate (DSS)-induced colitis in a mouse ulcerative colitis model [423].

In summary, IL-25, IL-33, and TSLP are released by epithelial cells at mucocutaneous surfaces and target similar cell populations to initiate and enhance type 2 immunity. Understanding the harmonization and functional distinctions of these epithelial cytokine pathways is crucial. These cytokines signal local inflammation and tissue damage, critical regulatory checkpoints for allergic inflammation at barrier surfaces. Targeting epithelial alarmins and type 2 inflammation has emerged as a promising therapeutic strategy for conditions such as asthma and chronic obstructive pulmonary disease (COPD). By focusing on antialarmins and next-generation biologics that can modulate epithelial responses, researchers have aimed to mitigate exacerbations and improve lung function in patients with type 2-high asthma [406, 424].

### Chemokines involved in type 2 immunity

By sensing the concentration gradient, chemokines are cytokines that attract immune cells to migrate toward the inflammatory site. Interactions between chemokines and chemokine receptors play crucial roles in recruiting primarily myeloid cells and Th2 cells in type 2 immunity. Eosinophils expressing the chemokine receptor CCR3 are attracted by the activation of the chemokine eotaxin family, which consists of eotaxin-1 (CCL11), eotaxin-2 (CCL24), and eotaxin-3 (CCL26), in allergic diseases [425]. CCL8, CCL-17, and CCL-24 produced by activated macrophages also induce the migration of eosinophils in an allergic airway disease mouse model [426]. Basophils are another essential myeloid cell type recruited to inflammatory sites during allergic reactions. Allergic reactions caused by exposure to peanuts in humans resulted in a significantly decreased number of circulating basophils with increased CCL2 levels, the source of which remains unidentified [427]. CCR2 is the dominant chemokine ligand for CD11b+ DC migration in allergic responses. In mouse models, CCR2 deficiency triggered the loss of half a subset of CD11b+ DCs, moDCs, after allergen exposure and resulted in less airway eosinophilia inflammation [428]. MoDCs produce proinflammatory chemokines after allergen challenge (CCL24, CCL2, CCL7, and CCL12), which are crucial for attracting eosinophils and monocytes to the target site [428]. CCL20 also activates immature DCs and promotes their maturation through binding to CCR6 in asthma [429]. More recently, the receptor CCR8 and its main ligand CCL1 were found to be involved in a mouse model of contact hypersensitivity. CCR8 can retain dendritic cells within the skin, which prevents them from migrating toward secondary lymphoid organs [430].

In asthma, Th2 cells are recruited to the airway through CCR4 due to the increased secretion of CCL22 by monocytes and macrophages, as well as CCL17 by bronchial epithelial cells and dendritic cells [431–433]. Moreover, CCL17 and CCL22, which are expressed mainly by keratinocytes, initiate the recruitment of Th2 lymphocytes via CCR4 in atopic dermatitis [434]. CCL18 induced chemotaxis and calcium flux in highly polarized human Th2 cells through CCR8 [435]. Increased expression of CCL8 in dermal dendritic cells and keratinocytes also promoted the development of atopic dermatitis by recruiting Th2 cells through CCR8, further amplifying eosinophilic inflammation [436, 437]. Moreover, both CCL18 and CCR8 are upregulated in patients with active EoE compared with subjects whose disease is in remission and normal controls [435]. The plasma levels of CXCL13 are significantly elevated and correlated with Tfh2 cells in patients with atopic asthma [438]. Moreover, exon array revealed that the transcription of CXCL13 was increased in asthma patients with CRSwNP and atopic dermatitis [439].

## Epithelial barrier theory and type 2 immunity

The epithelial barrier theory suggests that the recent rise in chronic noncommunicable diseases, including autoimmune and allergic disorders, is due to the disruption of epithelial barriers caused by exposure to harmful environmental agents [1]. Since the 1960s, over 350,000 chemicals have been introduced into our lives without adequate consideration of their effects on human and animal health. More than 110,000 of these chemicals have not been properly reported. The negative impacts of these substances on the body are continuously increasing due to changes in the human exposome—encompassing all environmental exposures, such as the diet, microbiome, and pollutants throughout an individual’s lifetime—driven by industrialization and modernization [357, 440, 441].

### Epithelial barrier defects due to exposure to barrier-damaging toxic substances and genetic reasons

The detrimental effects of exposure to compounds that damage epithelial barriers have been demonstrated through methods assessing both functional and molecular changes associated with reduced epithelial barrier integrity. These compounds cause direct cell death, metabolic and proinflammatory effects, and oxidative stress, disrupting the expression and structure of epithelial junction molecules. This disruption is evident either through changes in the expression levels of these junctional molecules or direct impairment of epithelial cells [442].

Epithelial barrier-damaging compounds, such as food emulsifiers, disrupt homeostasis in various ways, often triggering epithelitis characterized by the release of proinflammatory cytokines and damage to the epithelial barrier [22]. Detergent active substances such as sodium dodecyl sulfate (SDS) and similar surfactants induce significant inflammation, increasing ROS and IL-33 expression. These agents can cause eosinophilic inflammation in various tissues, increasing the expression of IL-33 and other proinflammatory factors [28, 443–445]. In a recent study, two commercial laundry detergents and two common surfactants (SDS and sodium dodecyl benzene sulfonate) were intranasally administered to mice [28]. Only four administrations once a day induced eosinophilic airway inflammation with increased IL-33 expression and activation of ILC2s. This inflammation was significantly reduced in Rag2-/- Il2rg-/- mice, which lack T and B cells, and in both ILC and Il33-/- mice. IL-5 reporter mouse experiments confirmed the role of IL-5 produced by ILC2s in facilitating eosinophilia. Detergent-induced IL-33 expression in airways was reduced by n-acetyl cysteine, an antioxidant, both in vivo and in vitro. Another study explored detergents and SDS in EoE and reported that common toothpastes contain high doses of SDS. Low doses of SDS decreased epithelial barrier integrity and increased IL-33 mRNA expression in cell lines and esophageal organoids. Mice exposed to very low doses of SDS (5 μg/ml) presented increased esophageal inflammation, IL-33, basal zone hyperplasia, CD4+ cell infiltration, and esophageal eosinophilia, indicating that detergents can trigger asthma and EoE [443].

Mutations in genes encoding essential epithelial barrier proteins are linked to asthma, AD, and food allergies [446–449]. Genetic polymorphisms in alarmin cytokines (TSLP and IL-33) and type 2 cytokines (IL-4 and IL-13) influence asthma risk and severity [448], although many asthma loci remain undiscovered [450]. Recent studies have highlighted the impact of epigenetic changes on epithelial barrier integrity [451]. The levels of histone deacetylases (HDACs) 1 and 9 and sirtuins (SIRTs) 6 and 7 are significantly increased in asthmatic bronchial epithelial cells [13, 452]. IL-4 and IL-13 increase HDAC and SIRT expression. HDAC inhibition improves barrier integrity by increasing TJ molecule synthesis in the asthmatic epithelium to the levels observed in control subjects.

Rare genetic variants impairing desmosome structure and barrier function were found in genes encoding desmoplakin and periplakin, contributing to EoE in 21% of multiplex families [453]. Mutations in the adapter-related protein complex 1 subunit sigma 1 (AP1S1) cause intestinal epithelial barrier defects, leading to congenital diarrhea [454]. Biallelic missense mutations in CLDN10B impair barrier integrity in eccrine sweat glands, causing mild ichthyosis and palmar hyperlinearity (HELIX syndrome) [455].

The role of filaggrin in asthma was explored through the sequencing of 109 asthma candidate genes [456]. Suppressed filaggrin and E-cadherin expression, along with increased IL-33 and TSLP, was observed in IL-4-treated bronchial epithelial cells. Filaggrin knockdown further downregulated E-cadherin and elevated IL-33 and TSLP when filaggrin was costimulated with IL-4, suggesting that the absence of filaggrin promotes Th2 inflammation, contributing to asthma progression.

### Relationship between the microbiome and epithelial barrier

#### Microbial dysbiosis in epithelial barrier-defective tissues

The colonization of opportunistic pathogens and loss of commensal bacteria followed by the translocation of bacteria to subepithelial areas are the main events that affect chronic inflammation and progression to disease in areas with leaky epithelial barriers.

Epithelial barrier damage caused by environmentally toxic compounds results in direct microbial dysbiosis [1]. Following barrier damage, the microbiota can translocate to subepithelial tissues [457]. Microbial translocation is known to cause several autoimmune and chronic diseases, such as Crohn’s disease [458], chronic HIV infection [459–461], SARS-CoV-2-related multisystem inflammatory syndrome [462], lupus [463] and fatty liver disease [464–466]. Owing to dysbiosis [467], some opportunistic pathogens, such as *Staphylococcus aureus* in AD patients, can colonize the affected tissue [468]. Abnormal interactions between the host microbiota and the epithelial barrier lead to abnormal mucosal immune responses, including the upregulation of Th17-, Th1-, and Th2-type responses; the downregulation of T regulatory cells; and dysregulated humoral immunity [469, 470]. Continuous exposure to harmful environmental factors can impair the metabolic flexibility of epithelial cells, affecting the regenerative capacity of intestinal tissue [471].

#### Immune response to commensals and opportunistic pathogens

The immune response to commensals and opportunistic pathogens has been observed as one of the reasons for microbial dysbiosis. Healthy individuals have host defense mechanisms that prevent opportunistic bacteria from causing infection. However, under pathological conditions such as chronic lung inflammation, bacteria can manipulate the immune response, allowing commensal bacteria to thrive and cause infections. In allergic airways, pathogenic bacteria evade the immune system by inactivating TLRs or becoming unrecognizable to TLRs. This reduces pathogen elimination, leading to infections. Dysregulation of gut microbial homeostasis, immunological modulation, and microbial interactions contribute to chronic illnesses [472, 473].

Neutrophils play a pivotal role in the defense against *Staphylococcus aureus* infections [474]. *S. aureus* targets neutrophils through various mechanisms, including chemotaxis, opsonophagocytosis, and neutrophil-mediated killing. The bacterium also kills host cells, including neutrophils, to evade the immune response. *S. aureus* has a large repertoire of immune evasion molecules that contribute to its survival after phagocytosis. These molecules include reactive oxygen species, nitric oxide, antimicrobial peptides, and neutrophil serine proteases. The extracellular adherence protein (Eap) and its homologs (EapH 1 and EapH 2) can noncovalently inhibit neutrophil serine proteases (NSPs) at low nanomolar concentrations, preventing bacterial killing and neutrophil lysis and the degradation of phenol-soluble modulins (PSMs) [475]. The PSMs target neutrophil formyl-receptor-2, which leads to degranulation and neutrophil lysis [475]. Additionally, NSPs can target and degrade staphylococcal immune evasion factors, including chemotaxis inhibitory protein of *S. aureus* (CHIPS) and staphylococcal complement inhibitor-A. The activity of these proteases varies against homologs of immune evasion proteins, possibly explaining the redundancy in the large number of immune evasion factors secreted by *S. aureus*. All three Eap proteins were shown to protect against the activity of NSPs against staphylococcal immune evasion factors [474, 475].

Diet-driven changes in the gut microbiome contribute to the increasing prevalence of food allergies. Fiber deprivation in mice led to an increase in the abundance of the bacterium *Akkermansia muciniphila*. The presence of *A. muciniphila* combined with fiber deprivation worsened food allergy symptoms by promoting anticommercial IgE coating and innate type 2 immune responses [476]. The succinate produced by the commensal *Trichomonas musculis* stimulates Tuft cell release of IL-25, which induces ILC2s to produce IL-13. This leads to increased Paneth cells and shifts in antimicrobial peptides, altering the bacterial microbiota composition and modulating intestinal bacterial homeostasis [477].

*Candida albicans* strains with high immune-cell-damaging capacity dominate the colonic mucosa of patients with inflammatory bowel disease. These strains aggravate intestinal inflammation through IL-1β-dependent mechanisms. Candidalysin, a toxin secreted by *C. albicans*, influences inflammatory immunity and Th17 cell antifungal responses.

The skin barrier plays a crucial role in protecting against environmental threats, including skin-resident microbes. Dysregulation of this barrier is a hallmark of AD and ichthyosis, leading to variable consequences for host immune control of colonizing commensals and opportunistic pathogens. An experimental study using murine models of AD and ichthyosis revealed that Malassezia, a common commensal fungus, grows excessively in AD-like skin due to structural and metabolic changes in the dysfunctional epidermal barrier environment [476, 478].

Mice with intestinal *Candida* dysbiosis showed enhanced Th2 responses after airway sensitization to house dust mites (HDMs), characterized by increased white cell and eosinophil counts in the airway and IgE concentrations in the serum. ILC2s were more abundant in the lungs, suggesting that ILC2s may mediate the enhanced Th2 response. This effect was not due to increased *Candida* in the lung, indicating gut‒lung axis interactions [479].

*Pseudomonas aeruginosa* induces a type 2 immune response, producing mucin that serves as an energy source. The toxin LasB, which is secreted by *P. aeruginosa*, processes and activates epithelial amphiregulin, inducing type 2 inflammation and mucin production. This “niche remodeling” by *P. aeruginosa* promotes colonization and allergic sensitization [480].

#### Mechanisms of decreased biodiversity and loss of commensal bacteria

A reduction in microbial diversity, often referred to as “biodiversity loss”, is considered one of the most common markers of gut dysbiosis. Loss of microbial diversity in the gut microbiome has been linked to many human diseases in Westernized countries [481]. Dysbiosis and biodiversity loss in the gut have been linked to common dermatological conditions such as atopic dermatitis and psoriasis. These findings suggest a potential connection between gut dysbiosis and skin health [482, 483]. The composition of the microbial community in the intestine can influence the functions of distant organs such as the brain, lung, and skin. This can lead to the co-occurrence of intestinal and skin diseases. Disruption of the dermis from skin wounds or the digestion of dermal hyaluronan results in increased expression of host defense genes in the colon, such as Reg3 and Muc2. Skin wounds also change the composition and behavior of intestinal bacteria. In vitro studies have demonstrated that the increased expression of Reg3 and Muc2 is induced by exposure to hyaluronan released by these skin interventions. The alteration of the colon microbiome after skin wounding is functionally significant, as these bacteria penetrate the intestinal epithelium and exacerbate DSS-induced colitis. This evidence shows the existence of a skin‒gut axis, whereby damage to the skin disrupts intestinal homeostasis and alters the gut microbiome [484].

Neutrophils are crucial for periodontium defense. In mice lacking CXCR2, which recruits neutrophils, there is a significant alteration in the composition of the periodontal microbiome, resulting in spontaneous inflammatory bone loss. The presence of active CXCR2 neutrophil receptors was able to reestablish the gingival tissue microbiota [485].

The deletion of TLR4 in the gut epithelium has been linked to exacerbated intestinal and pancreatic injury during acute pancreatitis (AP) [486]. This is believed to be due to gut dysbiosis and Paneth cell dysfunction. Interestingly, *Lactobacillus reuteri* was found to activate Paneth cells and promote epithelial proliferation, suggesting that Lactobacillus supplementation could represent a potential therapeutic approach for managing AP.

rTsSPI, a serine protease inhibitor from *Trichinella spiralis*, has been demonstrated to possess therapeutic potential to induce a Th2-type response, reducing neutrophil recruitment in the colonic lamina propria and TNF-α levels in the colon. rTsSPI also increases M2 macrophage recruitment, IL-10 expression, and the expression of adhesion molecules. Furthermore, it enhances gut microbiota diversity and increases the abundance of *Bifidobacterium* and *Ruminoclostridium* [487].

*Staphylococcus aureus* is the most abundant bacterium that colonizes tissues with barrier damage to the skin and upper respiratory mucosa. When *S. aureus* breaches the epithelial barrier, it has been linked to asthma, CRS, and AD, and high levels of IgE antibodies specific to *S. aureus* antigens are correlated with increased disease severity and exacerbation in affected individuals [488–492]. In addition to *S. aureus*, facultative pathogens such as *Streptococcus pneumoniae*, *Haemophilus influenzae*, and *Moraxella catarrhalis* have also been associated with asthma development [493–495]. The overgrowth of these pathogens reduces the local biodiversity of the microbiome, which may contribute to the development of allergic diseases, as suggested by the hygiene hypothesis.

### Inflammation in the epithelium and chronicity

A transgenic mouse model (EoE33) overexpressing esophageal IL-33 exhibited EoE-like pathology, including inflammation, eosinophilia, and Th2 responses. This effect was accompanied by increased levels of type 2 cytokines and immune/remodeling molecules, suggesting that IL-33 overexpression may induce EoE-like immunopathology by activating the IL-13 pathway [496].

Commonly used household detergents have been shown to disrupt airway, skin and EoE epithelial barriers [17, 23, 29]. Professional and household dishwashing detergents and rinse agents also cause barrier disruption and induce inflammatory responses in epithelial cells. Transcriptomic and proteomic data show upregulation of cell death, development, metabolism, proliferation, and immune and inflammatory responses in epithelial cells [21].

Exposure to air pollutants activates type 2 inflammatory pathways involved in asthma pathogenesis, promoting the release of epithelial cytokines that drive Th2 responses, such as IL33 and TSLP, in human bronchial epithelial cells, which can induce Th2 cytokine synthesis. In addition, pollutants can induce oxidative stress and the production of proinflammatory cytokines, such as IL-6, IL-8 and IL-1B [497].

In chronic rhinosinusitis with nasal polyps, the inflammatory cytokines IL-4, IL-12, and IL-5 drive type 2 inflammation, recruiting and activating immune cells. These mechanisms lead to structural changes, including polyp formation, tissue remodeling, and chronic barrier dysfunction [498].

Aeroallergen exposure induces the extracellular release of ADP and ATP nucleotides, which activate P2Y1-Rs, which induce the translocation and release of IL-33 and HMGB1 from airway epithelial cells. P2Y13-R blockade attenuates asthma onset and severity in a chronic asthma model [499].

### Epigenetic regulation of defective epithelial barriers in chronic type 2 inflammation

Epigenetic mechanisms, particularly histone modifications such as acetylation and deacetylation, play crucial roles in regulating epithelial barrier integrity by controlling the expression of genes essential for barrier function, such as those encoding tight junction proteins [13, 452]. Proinflammatory cytokines exhibit reduced methylation levels, whereas regulatory cytokines, including IL10RA and TGFBR2, show increased methylation [452]. Epigenetic changes also influence the healing capacity of epithelial barriers, as evidenced by the impaired ability of epithelial stem cells from defective areas to form strong tight junctions—a condition that can be ameliorated through histone deacetylase inhibition [13]. Furthermore, epigenetic regulation contributes significantly to the chronicity and persistence of epithelial barrier defects, where continuous epigenetic alterations can result in ongoing barrier dysfunction, potentially leading to chronic inflammatory conditions [13, 452].

**Fig. 9:**
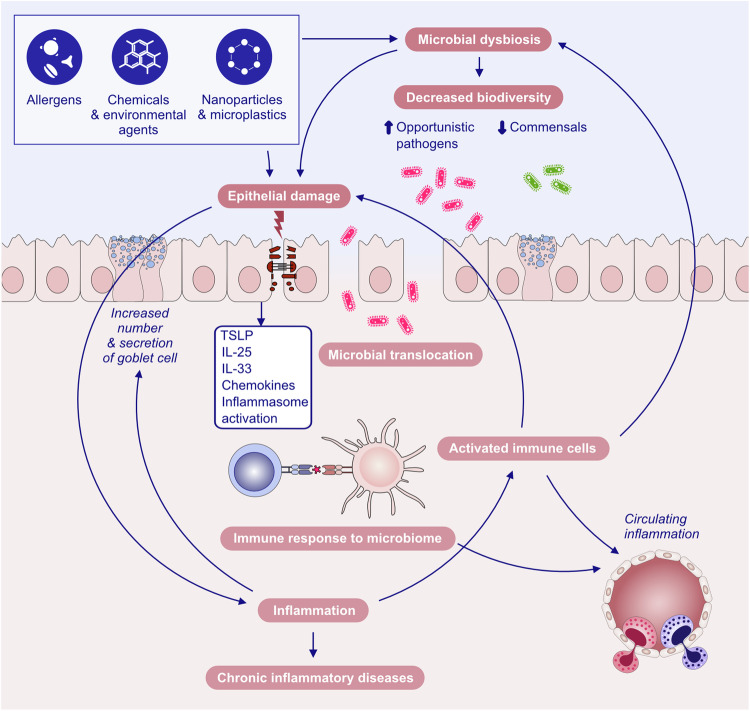
**Overview of the epithelial barrier theory**. Epithelial inflammation, epithelitis, and opportunistic pathogen colonization are caused by epithelial barrier dysfunction induced by genetic deficiencies in barrier molecules or exposure to environmentally toxic substances. A healthy epithelial barrier is linked to high microbiome diversity. Microbial dysbiosis and the translocation of commensal and opportunistic pathogens across epithelial barriers increase alarmin and chemokine production and alter the activation thresholds of cells and migrating immune cells. This leads to an inflammatory state that contributes to allergic, autoimmune, and metabolic diseases. The inability of the epithelium to fully repair and close the barrier perpetuates a vicious cycle of leaky barriers, microbial dysbiosis, and chronic inflammation. Individuals with barrier dysfunction exhibit elevated levels of proinflammatory cytokines and chemokines in the circulation, further exacerbating systemic and chronic inflammation

An unhealed epithelial barrier results in ongoing epithelitis, triggering localized or sometimes systemic inflammatory responses. This vicious cycle of interconnected events leads to persistent peri-epithelial inflammation and barrier leakiness (Fig. 9).

## Inborn Errors of Immunity and Type 2 Response

Inborn errors of immunity (IEIs) provide new insights into how the immune system works and include nearly 500 genetic causes and counts [500]. The initial understanding of IEI, which considers IEI predominantly in relation to its predisposition to infections, but in light of recent research, we now recognize its role in inflammation, cancer surveillance, autoimmunity and allergies. Monogenic IEI with excessive type 2 responses can be particularly difficult to distinguish from severe atopic disorders, which led to the proposal of the term “primary atopic disorders” (PAD) [501]. These PADs can be further subdivided into the following categories to focus on mechanisms, although there may be overlap.

### Immune deficiencies related to altered T-cell signaling

T-cell receptor (TCR) signaling, cytokine signaling pathways and defects in the actin cytoskeleton can impair TCR signaling and lead to excessive type 2 responses. The CARD11–BCL10–MALT1 (CBM) complex transduces signals from T and B-cell receptors to the nuclear factor-kappa B (NF-kB) and mTOR signaling pathways [502]. Dominant-negative mutations causing loss of function in Caspase Recruitment Domain Family Member 11 (CARD11) and biallelic variants in mucosa-associated lymphoid tissue lymphoma translocation protein 1 (MALT1) can lead to combined immune deficiency and atopic disorders, mainly severe AD [502, 503]. Possible explanations for the atopic phenotype observed in these defects include impaired NF-kB signaling; this is supported by patients with NF-kB essential modulator (NEMO) and NFKB2 defects showing atopic features and impaired regulation of mTOR1, which is involved in the function of Tregs [502, 503]. Capping protein regulator and myosin 1 linker 2 (CARMIL2) is another key molecule involved in CD28-mediated T-cell activation and is required for NF-kB signaling. CARMIL2 deficiency can cause a wide range of symptoms, including recurrent infections, inflammatory bowel disease, Epstein–Barr virus (EBV)-related smooth muscle tumors (SMTs) and atopic diseases. A reduced number and function of Tregs have also been documented in these patients [504].

Formerly known as Job syndrome, dominant negative mutations in signal transducer and activator 3 (STAT3) were among the first IEIs associated with the type 2 immune response [505]. Early-onset dermatitis, recurrent pulmonary infections complicated with pneumatocel formation, chronic mucocutaneous candidiasis, the absence of acute phase reactions, and eosinophilia along with elevated IgE levels are the main clinical manifestations. The presence of nonimmunologic findings, including dental, skeletal and vascular abnormalities, is a distinct feature of IL-6/STAT3-related diseases [506]. An excessive Th2 response has been documented in newly described IL6R-, IL6ST-, ZNF341- and ERBIN-related IEIs involved in the IL-6/STAT3 signaling pathway. These patients may also have severe atopic dermatitis, asthma and eosinophilic gastrointestinal disorders. Interestingly, anaphylaxis due to defective mast cell degranulation is less likely to occur with defective STAT3 signaling. The nonimmunologic features may depend on pathway disruption, especially when IL-11 and leukemia inhibitory factor [507] are involved, which are upstream of the STAT3 molecule [507]. Defective IL-21 signaling and ERBIN-mediated defective inhibition of GATA3 transcription leading to an enhanced IL-4 response are proposed mechanisms for increased IgE and atopic features in DN STAT3 deficiency [507]. Recently, a few groups reported that gain-of-function variants in the STAT6 gene, the key transcription factor mediating IL-4-related biological pathways, constitute the perfect model for Th2 disorders. Early-onset severe atopic features, including anaphylaxis, severe asthma and eosinophilic gastrointestinal disorders, are prominent in affected patients. Dupilumab and JAK inhibitors are well tolerated and effective at controlling atopic features [508].

**Fig. 10:**
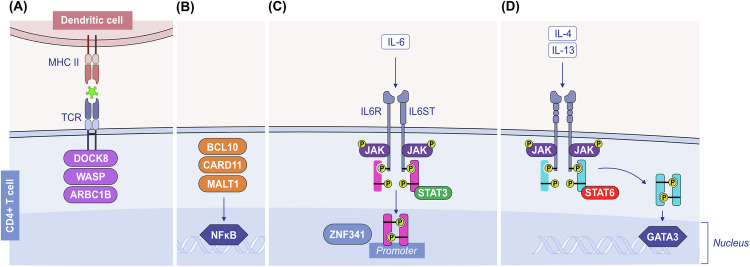
**Pathways involved in inborn errors of immunity with a Type 2 response**. **A** Genes involved in the regulation of the actin cytoskeleton. **B** Role of the BCM complex in transducing signals to the NFKB pathway. **C** IL-6 cytokine family and STAT3-related pathways. **D** IL-4- and IL-13-related STAT6 signaling. ARBC1B; Actin-related protein 2/3 complex, subunit 1B, BCL-10 B-cell CLL/lymphoma 10, CARD-11 Caspase Recruitment Domain Family Member 11, DOCK8; Dedicator of Cytokinesis 8, GATA3 GATA-binding protein 3, IL6R Interleukin 6 receptor, IL6ST Interleukin 6 cytokine family signal transducer, JAK Janus kinase, MALT-1 Mucosa-associated lymphoid tissue lymphoma translocation protein 1, MHC-II Major histocompatibility Complex II, NFKB Nuclear factor-kappa B, STAT Signal transducer and activator, TCR T-cell receptor, WASP Wiskott–Aldrich syndrome protein, ZNF341 Zinc finger protein 341

The actin cytoskeleton is a dynamic network involved in various cellular functions, such as the formation of immune synapses, cell migration, proliferation and differentiation. Unsurprisingly, its proper functioning requires tight regulation. Wiskott–Aldrich syndrome (WAS) is caused by mutations in the WAS protein (WASp), which plays a central role in actin polymerization, with Dedicator of Cytokinesis 8 (DOCK8), a guanine nucleotide exchange factor, and the Actin-Related Protein 2/3 (ARP2/3) complex [509]. Along with autosomal recessive DOCK8 and actin-related protein 2/3 complex subunit 1B (ARBC1B) deficiencies, WAS may lead to recurrent infections, severe eczema, asthma, food allergies and autoimmunity. Associated thrombocytopenia may distinguish WAS and ARBC1B deficiency from DOCK8 deficiency [509] (Fig. 10).

### Decreased T-cell repertoire diversity and immune deficiencies

Severe combined immune deficiency (SCID) is the most severe form of IEI and can lead to death if left untreated. Owing to the very low percentage of T cells/absent T cells, no Th2 response is expected. We now know that the degree of expression of some variants can lead to a spectrum ranging from the severe form of SCID to the late-onset and milder “leaky SCID” or Omenn syndrome. Autologous and oligoclonal T-cell expansion with a restricted TCR repertoire, as occurs in Omenn syndrome, leads to infiltration and damage of several organs, particularly the skin and intestine, and manifests as dermatitis, diarrhea, lymphadenopathy and hepatosplenomegaly [510]. Like in typical type 2 diseases, laboratory tests can reveal hypereosinophilia with elevated IgE levels. In mouse models, the limited repertoire results in a preference for the Th2 response [511]. Patients may require immunosuppressants, which can lead to further complications due to their susceptibility to infections. The only curative treatment option is HSCT [510].

### Immune deficiencies with defective regulatory T-cell development/function

IPEX syndrome (immunodysregulation-polyendocrinopathy-enteropathy x-linked) is caused by hemizygous mutations in the FOXP3 gene, which encodes the basic transcription factor of regulatory T cells. Severe atopic dermatitis, persistent diarrhea and autoimmunity, as in type 1 diabetes, are characteristic symptoms. The course of the disease is fatal, although there are also milder cases with “atypical” or “late-onset” forms. Most patients require immunosuppressive or immunomodulatory treatments, with hematopoietic stem cell transplantation (HSCT) being the only potentially curative treatment. In IPEX syndrome, other genetic defects affecting the development or function of Tregs, such as IL2RA, LRBA, CTLA4, BACH2, STAT3 gain-of-function (GOF), IL10, DEF6, FERMT1, IL2RB and IKAROS GOF, have been identified. These Tregopathies can have predominantly autoimmune and inflammatory manifestations, but allergic symptoms are less pronounced than those associated with IPEX syndrome [500, 512].

## Conclusion

Major discoveries in recent decades in the area of the type 2 immune response have improved our understanding of the pathogenesis of many diseases and novel treatment modalities. They can provide a better understanding of the involved cells and their subsets; the discovery of type 2 innate lymphoid cells, eosinophil subsets, novel mast cell receptors, and neuroimmune regulation; the demonstration of epithelial barrier defects as the initiators of type responses induced by environmental and lifestyle changes; the increased prevalence of many type 2 diseases with multimorbidities; and the discovery and successful clinical use of biologics that specifically target the IgE, IL-4/IL-13, IL-5 and TSLP pathways and JAK inhibitors.

Continuous research and innovation are necessary to develop treatments that address the unmet needs of patients with Th2-driven diseases, advance precision medicine and improve the quality of life for affected individuals. Understanding type 2 immune mechanisms is crucial for improving the management of asthma and AD through precision medicine and biomarker discovery. Continued research will advance treatment strategies and patient outcomes for these complex conditions.
